# Characterization of ecotin homologs from *Campylobacter rectus* and *Campylobacter showae*

**DOI:** 10.1371/journal.pone.0244031

**Published:** 2020-12-30

**Authors:** Cody Thomas, Harald Nothaft, Ruchi Yadav, Christopher Fodor, Abofu Alemka, Oluwadamilola Oni, Michael Bell, Balázs Rada, Christine M. Szymanski

**Affiliations:** 1 Department of Microbiology and Complex Carbohydrate Research Center, University of Georgia, Athens, Georgia, United States of America; 2 Department of Biological Sciences, University of Alberta, Edmonton, Alberta, Canada; 3 Department of Infectious Diseases, University of Georgia, Athens, Georgia, United States of America; CINVESTAV-IPN, MEXICO

## Abstract

Ecotin, first described in *Escherichia coli*, is a potent inhibitor of a broad range of serine proteases including those typically released by the innate immune system such as neutrophil elastase (NE). Here we describe the identification of ecotin orthologs in various *Campylobacter* species, including *Campylobacter rectus* and *Campylobacter showae* residing in the oral cavity and implicated in the development and progression of periodontal disease in humans. To investigate the function of these ecotins *in vitro*, the orthologs from *C*. *rectus* and *C*. *showae* were recombinantly expressed and purified from *E*. *coli*. Using CmeA degradation/protection assays, fluorescence resonance energy transfer and NE activity assays, we found that ecotins from *C*. *rectus* and *C*. *showae* inhibit NE, factor Xa and trypsin, but not the *Campylobacter jejuni* serine protease HtrA or its ortholog in *E*. *coli*, DegP. To further evaluate ecotin function *in vivo*, an *E*. *coli* ecotin-deficient mutant was complemented with the *C*. *rectus* and *C*. *showae* homologs. Using a neutrophil killing assay, we demonstrate that the low survival rate of the *E*. *coli* ecotin-deficient mutant can be rescued upon expression of ecotins from *C*. *rectus* and *C*. *showae*. In addition, the *C*. *rectus* and *C*. *showae* ecotins partially compensate for loss of N-glycosylation and increased protease susceptibility in the related pathogen, *Campylobacter jejuni*, thus implicating a similar role for these proteins in the native host to cope with the protease-rich environment of the oral cavity.

## Introduction

Bacteria have developed various strategies to survive in their ecological niche. In the human host, proteases are vital players of the immune system for protecting against and clearing pathogens [1]. One of these particular areas is the oral cavity where polymorphonuclear neutrophils (PMNs) of the innate immune system are the first line of defense against invading microorganisms. PMNs are the most prominent circulating leukocytes in humans [2] and contain a variety of neutrophil serine proteases (NSPs), the predominant one being neutrophil elastase (NE). Neutrophils can engulf invading bacteria via phagocytosis to form a phagolysosome where microbial killing takes place by the action of these proteases, reactive oxygen species and additional antimicrobial mechanisms. Alternatively, neutrophils can also release these proteases at inflammatory sites during activation, particularly in neutrophil extracellular traps (NETs) [3]. Aside from assisting in pathogen destruction, NETs and NSPs are also involved in human inflammatory conditions including chronic lung diseases like cystic fibrosis [4–8]. Here, overstimulation e.g. by *Pseudomonas aeruginosa* or delayed apoptosis of neutrophils results in excessive accumulation of NETs and NSPs and in subsequent lung tissue degradation [8,9].

Bacterial strategies to avoid neutrophil-mediated killing include launching a general survival response, avoiding contact, preventing phagocytosis, surviving inside the neutrophil, and inducing cell death [10,11]. Specific countermeasures against the action of proteases are the production of protease inhibitors, including serine protease inhibitors such as ecotin [12], or masking proteolytic cleavage sites through glycosylation of protease target proteins [13]. Protein glycosylation is the most common post-translational modification in nature and exists in all three domains of life. The foodborne pathogen, *Campylobacter jejuni*, was the first bacterium demonstrated to possess a general N-linked protein glycosylation (*pgl*) system [14] that adds a unique heptasaccharide to greater than 80 non-cytoplasmic proteins [15,16] and this modification has been shown to protect *C*. *jejuni* surface proteins from the action of chicken gut proteases [13]. When incubated with chicken cecal contents that contain a variety of gut proteases, a *C*. *jejuni* mutant defective in N-glycosylation showed significantly lower survival rates when compared to the wild-type strain, a phenotype that could be rescued by the addition of a protease inhibitor cocktail [13]. Whilst the *pgl* locus in *C*. *jejuni* exclusively harbors genes required for the biosynthesis of the N-linked oligosaccharide, further examination of *pgl* loci from other *Campylobacter* species [17] revealed the presence of an open reading frame for a generic serine protease inhibitor, an ecotin homolog of *E*. *coli*. Interestingly this ecotin homolog was present in specific non-thermophilic *Campylobacters* primarily described to inhabit the oral cavity and associated with the onset of periodontitis [18], an inflammatory disease caused by a consortium of microbes and host defense mechanisms. Here, bacterial killing by neutrophils comes as a double-edged sword; reactive oxygen species and bactericidal proteins such as elastase not only neutralize bacteria, but also damage host tissues and increase the severity and progression of periodontal diseases [19]. *C*. *rectus*, in particular, is a pathogen detected at elevated levels in diseased human subgingival sites when compared to healthy non-diseased controls [20–23] and is often associated with other oral pathogens such as *Porphyromonas gingivalis* [24]. Other *Campylobacter* species isolated from oral sites include *Campylobacter showae*, *Campylobacter curvus*, *Campylobacter concisus*, *Campylobacter sputorum* and *Campylobacter hominis* [20–22,25]. In these *Campylobacter* species, the N-glycan is also believed to protect glycoproteins from proteolysis, however, ecotin might provide an additional survival advantage in the periodontal pockets that contain high levels of neutrophil serine proteases [26,27].

Ecotin was first described in *E*. *coli* as a periplasmic protease inhibitor that exhibits a broad specificity toward exogenous serine proteases including trypsin, chymotrypsin, factor Xa, NE, kallikrein, urokinase and factor XII, but not against metallo-, aspartyl and sulfhydryl proteases, or its own proteases [28–32]. Ecotin homologs have since been found in more than 300 organisms, predominantly in Gram-negative bacterial pathogens such as *Pseudomonas aeruginosa*, *Shigella flexneri*, *Yersinia pestis*, *Burkholderia pseudomallei* and *Klebsiella pneumoniae* [12,31–33], but also in two eukaryotic protozoan parasites within the *Trypanosomatidae* genus (*Leishmania major* and *Trypanosoma cruzi* [12,34,35]), and in some plant pathogens e. g. *Pantoea citrea* [31]. In the latter case, ecotin was found to be less potent in binding to NE when compared to the *E*. *coli* homolog, suggesting that the protein may have evolved to recognize alternate proteases specific to its host [31]. A novel feature of ecotin was recently described in *P*. *aeruginosa* [36]. Here, ecotin was shown to escape the cell and bind to biofilm matrix exopolysaccharides (PsI) [36] that are known to protect bacterial communities against antimicrobial proteins [37]. Although it is unknown whether *P*. *aeruginosa* ecotin is secreted through a non-classical secretion pathway [38] or through cell lysis [39], it has been shown that ecotin enhances the persistence and survival of *P*. *aeruginosa* in biofilms commonly found in the airways of cystic fibrosis patients [36].

In this study, we investigated if ecotin homologs from *Campylobacter* species share broad serine protease specificity. We tested *C*. *rectus* and *C*. *showae* ecotin homologs against a panel of proteases in a newly developed fluorescence-based peptide degradation assay and found that the *Campylobacter* ecotins inhibit the activities of elastase, factor Xa and trypsin. However, protein degradation-based assays showed that the periplasmic bacterial serine proteases, DegP from *E*. *coli* and the DegP homolog, HtrA from *C*. *jejuni*, are not affected by the addition of ecotin. *C*. *rectus* and *C*. *showae* ecotins partially rescue the protease-sensitive phenotype of a *C*. *jejuni* N-glycosylation mutant. In addition, cell killing assays demonstrated that an ecotin-deficient *E*. *coli* strain expressing ecotin homologs from *C*. *showae* and *C*. *rectus* showed comparable survival rates to the *E*. *coli ecotin-*complement when incubated with live human neutrophils or purified NETs, suggesting that the *Campylobacter* ecotin homologs fulfill a similar function in the native host.

## Materials and methods

### Bacterial strains, plasmids and growth conditions

Bacterial strains and plasmids used in this study are listed in Table 1. *Escherichia coli* was grown on LB agar or in 2xYT broth at 37°C with shaking at 220 rpm. *Campylobacter* strains used as a source for chromosomal DNA were *Campylobacter rectus*, *Campylobacter showae* (grown on Blood Heart Infusion (BHI) supplemented with 5% horse blood under anaerobic conditions) and *C*. *jejuni* 11168 (grown on MH under microaerophilic conditions). The antibiotics ampicillin (100 μg/mL), chloramphenicol (25 μg/mL) and kanamycin (25 μg/mL) were added to the growth medium when needed for selection.

**Table 1:** Bacterial strains and plasmids used in this study.

Strain or plasmid	Characteristics (genotype, description or source)	Reference
***E*. *coli***		
DH5α	F^–^ *endA1* *glnV44* *thi-1* *recA1* *relA1* *gyrA96* *deoR nupG* *purB20* φ80d*lacZ*ΔM15 Δ(*lacZYA-argF*)U169, hsdR17(*r*_*K*_^–^*m*_*K*_^+^), λ^–^	Invitrogen
BL21(DE3)	*E*. *coli* str. B F^–^ *ompT* *gal* *dcm* *lon* *hsdS*_*B*_(*r*_*B*_^–^*m*_*B*_^–^) λ(DE3 [*lacI* *lacUV5*-*T7p07* *ind1* *sam7* *nin5*]) [*malB*^+^]_K-12_(λ^S^)	Invitrogen
C600 (RK212.2)	*leu thr thi lacy supE44 tonA;* pRK212.2, Amp^R^, Tet^R^	[40]
***Campylobacter***		
*C*. *rectus*	Wildtype strain, ATCC 33238; human periodontal pocket	[20]
*C*. *showae*	Wildtype strain, CCUG 30254; human gingival crevice	[25]
*C*. *jejuni* 11168	Wildtype strain, clinical isolate used for genome sequencing	[41]
*C*. *jejuni* 81–176	Clinical isolate, wildtype strain	[42]
*C*. *jejuni* 81–176 (*pglB*)	*C*. *jejuni* 81–176 *pglB*::*kan*	[14]
**Plasmids**		
pET22b	*E*. *coli* expression vector, *pelB* signal sequence, C-terminal His_6_-Tag sequence, IPTG-inducible, Amp^R^	Novagen
pWA2	Expression of soluble periplasmic His_6_-tagged *Cj*-CmeA under control of Tet promoter in pBR322, Amp^R^	[43]
pET22b(*pelB*-ecotin-*Cr*)	pET22b expressing His_6_-tagged *C*. *rectus* ecotin with *pelB* leader peptide, Amp^R^	This study
pET22b(*pelB*-ecotin-*Csh*)	pET22b expressing His_6_-tagged *C*. *showae* ecotin with *pelB* leader peptide, Amp^R^	This study
pET22b(ecotin-*Ec)*	pET22b expressing native His_6_-tagged *E*. *coli* ecotin, Amp^R^	This study
pET22b(ecotin-*Cr*)	pET22b expressing native His_6_-tagged *C*. *rectus* ecotin, Amp^R^	This study
pET22b(ecotin-*Csh*)	pET22b expressing native His_6_-tagged *C*. *showae* ecotin, Amp^R^	This study
pCE111-28(*pelB* ecotin-*Cr*)	pCE111-28 expressing His_6_-tagged *C*. *rectus* ecotin with *pelB* leader peptide, Cm^R^	This study
pCE111-28(*pelB*-ecotin-*Csh*)	pCE111-28 expressing His_6_-tagged *C*. *showae* ecotin with *pelB* leader peptide, Cm^R^	This study
pCE111-28(ecotin-*Cr*)	pCE111-28 expressing native His_6_-tagged *C*. *rectus* ecotin, Cm^R^	This study
pCE111-28(ecotin-*Csh*)	pCE111-28 expressing native His_6_-tagged *C*. *showae* ecotin, Cm^R^	This study
pCE111-28(ecotin-*Ec)*	pET22b expressing native His_6_-tagged *E*. *coli* ecotin, Cm^R^	This study
pCE111-28	*C*. *jejuni* expression vector, plasmid pRY111 with σ^28^ promoter of *flaA*, Cm^R^	[44]
pKD4	FRT flanked kan gene, template plasmid for mutagenesis Kan^R^, Amp^R^	[45]
pKD46	λ Red recombinase (γ, β, and exo from λ phage), *ara*C-*Para*B, Amp^R^	[45]
pSC20	Modified pQE60 plasmid expressing His_6_-tagged DegP from *E*. *coli*, IPTG inducible, Amp^R^, Cm^R^	[46]
pET22b/htrA	pET22b expressing His_6_-tagged HtrA from *C*. *jejuni*, Kan^R^	This study

### Construction of plasmids

Ecotin genes from *E*. *coli*, *C*. *rectus* and *C*. *showae* were PCR-amplified from chromosomal DNA with the respective oligonucleotides (S1 Table). Obtained PCR products were purified and digested with the respective restriction nucleases and ligated into plasmid pET22b digested with the same enzymes (S1 Table). After transformation of *E*. *coli* DH5α, positive clones were identified by plasmid-restriction analysis and further confirmed by DNA sequencing. On these constructs, ecotin proteins are expressed as C-terminal 6xHis-tagged (His_6_) fusion proteins. In the case of *Campylobacter* ecotins, the respective genes were cloned with either their native signal peptide or, the native signal sequence as determined by SignalP [47] was replaced with the *pelB* leader peptide present on plasmid pET22b. Ecotin from *E*. *coli* was expressed with its native signal sequence.

For expression in *Campylobacter*, the ecotin genes were amplified by PCR using the pET22b derivatives as template in combination with oligonucleotides hybridizing upstream of the ribosomal binding site and down-stream of the His_6_-coding sequence. Obtained products were digested with the respective restriction nucleases (S1 Table) and ligated into the corresponding sites on the *E*. *coli*-*Campylobacter* shuttle vector pCE111-28. Verified constructs were mobilized into *Campylobacter* using *E*. *coli* C600 (RK212.2) as previously described [48,49]. Whole cell lysates of *C*. *jejuni* were prepared as described [49].

The *htrA* gene (including its native periplasmic secretion signal peptide) was amplified from chromosomal DNA of *C*. *jejuni* with oligonucleotides htrA-NdeI-F and htrA-XhoI-R. Obtained, purified and NdeI-XhoI digested PCR product was ligated into plasmid pET22b digested with the same enzymes. After transformation of *E*. *coli* DH5α plasmids isolated from selected colonies were analysed by DNA restriction and confirmed by DNA sequencing. On this construct *Cj*-HtrA is C-terminally fused to a His_6_ sequence. One positive plasmid candidate was used to transform *E*. *coli* BL21 for *Cj*-HtrA expression and purification.

### Construction of *E*. *coli* ecotin mutant (*Ec eco*::*kan*)

The *E*. *coli* BL21 ecotin deletion mutant (*Ec eco*::*kan*) was constructed following the protocol of Wanner and Datsenko [45]. Briefly, the kanamycin (*kan*) cassette from plasmid pKD4 was PCR amplified with oligonucleotides pKD4-ecotin-F and pKD4-ecotin-R. The purified PCR product was then electroporated into *E*. *coli* BL21 carrying plasmid pKD46 grown in 2xYT + 1% arabinose. After out-growth for 1 h at 30°C cells were spread on LB-kan agar and grown at 37°C. Candidate colonies (*eco*::*kan* = KanR, AmpS) that have the ecotin gene replaced with the kan cassette by simultaneously having lost the temperature-sensitive plasmid pKD46 were confirmed by PCR analysis of their chromosomal DNA with oligonucleotides EC-ecotin-F and EC-ecotin-R that hybridize outside of the recombination event. One candidate from which the correct PCR product with a size of 2119 bp was obtained was used for further analyses.

### Expression and purification of proteins in *E*. *coli*

*Ecotin proteins*: *E*. *coli* BL21 containing pET22b-ecotin expression plasmids were grown in 4 ml overnight culture, used to inoculate fresh growth medium to an OD_600_ of 0.1 and grown until an OD_600_ of 0.6 was reached. Ecotin expression was induced by the addition of Isopropyl β-D-1-thiogalactopyranoside (IPTG) to a final concentration of 0.3 mM. Cells were further grown overnight (18 h) and harvested by centrifugation 8,000 x g at 4°C. Cells were re-suspended in PBS and passed through a homogenizer (EmulsiFlex-C5, Avestin at 10000 PSI for 5 minutes). Obtained cell lysates were centrifuged at 16,000 xg for 30 minutes at 4°C. The resulting supernatant was run through Ni-NTA column washed 3 times with 15 ml of 15 mM imidazole and bound ecotin proteins were eluted with 6 column volumes (6 x 1 ml) 300 mM imidazole-PBS. Aliquots of elution fractions were analyzed by a 12.5% SDS-PAGE. Fractions that contain ecotin were dialyzed against 4 l PBS at 4°C overnight with PBS changed after 12 h. Purified ecotin proteins were stored at 4°C until further use.

*CmeA-His*_*6*_: The *C*. *jejuni* CmeA-His_6_ protein was expressed from pMW2 and purified as previously described [43].

*HtrA-His*_*6*_ *and DegP-His*_*6*_: HtrA-His_6_ was expressed and purified from *E*. *coli* BL21/pET22b-htrA-His_6_ grown in LB broth at 37°C to an OD_600_ of 0.6. HtrA-His_6_ expression was induced with 500 mM IPTG for 5 h. HtrA-His_6_ was purified and stored as described above for the ecotin proteins. DegP-His_6_ overexpression and purification from plasmid pCS20 was performed as previously described [46].

### Complementation of the *E*. *coli eco*::*kan* mutant

The *E*. *coli* BL21 *eco*::*kan* mutant was transformed with the pET22b-ecotin expression plasmids by electroporation [50]. After selection on LB amp plates select colonies were inoculated and grown in 10 mL of LB broth at 37°C to an OD_600_ of 0.6 and induced with IPTG. IPTG concentrations used to induce ecotin expression were: *Ec-ecotin* = 0.1 mM, *Cr-ecotin* = 0.4 mM and *Csh-ecotin* = 0.4 mM. Cultures were grown for an additional 4 h and cells were harvested by centrifugation at 8000 xg for 30 minutes at 4°C. Cells were re-suspended in ice-cold 1 x PBS and directly used in the bacterial neutrophil killing assay with intact neutrophils.

### Chicken cecal content (CCC) protease protection assays

The effect of *C*. *rectus* and *C*. *showae* ecotins on the viability of *C*. *jejuni* wildtype and *pglB* mutant in medium supplemented with chicken cecal contents (CCC) was investigated as described [13] using chicken cecal samples from 1-week old chickens obtained from the Poultry Research Facility, Department of Agriculture, Food and Nutritional Science, University of Alberta. CCC were obtained from animal studies carried out in accordance with the protocol approved by the Animal Care and Use Committee at the University of Alberta.

### Trypsin serine protease protection assay

CmeA-His_6_ from *C*. *jejuni* was used as protein substrate. CmeA-His_6_ (10 nM) was incubated with ecotin (15 nM) or PBS (control) and mixed with (10 nM) of trypsin (GIBCO®). Samples were incubated at 37°C or 45°C with 15 μl aliquots taken every 60 min. Aliquots were immediately mixed with protein loading dye, heated for 5 min at 95°C and kept on ice for at least 5 min before samples were analyzed by 15% SDS-PAGE followed by Coomassie staining.

### Self-protease HtrA and DegP assay

Soluble, CmeA-His_6_ from *C*. *jejuni* was used as the proteolytic substrate. The assay contained CmeA-His_6_ (10 nM), DegP-His_6_ or HtrA-His_6_ (1 nM) and ecotin from either *E*. *coli*, *C*. *rectus* or *C*. *showae* (15 nM) or an equal volume of PBS as a negative control in a total volume of 150 μl. Samples were initially mixed on ice and then incubated at 45°C. Aliquots of 15 μl were taken after 0, 1, 3, 6 h of incubation, immediately mixed with protein loading dye, incubated for 5 min at 95°C and stored at -20°C until samples were analyzed by 12.5% SDS-PAGE followed by Coomassie staining.

### FRET assay

Our approach uses a terminally labelled fluorophore/quencher peptide (Dabcyl-DQNATIDGRKQ-Edans) with Edans-fluorophore and Dabcyl-quencher and a factor Xa protease cleavage site (IDGR). When factor Xa cleaves the peptide, the fluorophore becomes spatially separated from the quencher resulting in increased levels of fluorescence (Fig 4A), whereas upon inactivation or in the absence of factor Xa little to no fluorescence should be observed. To determine the optimal ecotin to protease ratio and reaction time, the FRET peptide was incubated with a constant amount of factor Xa and with increasing amounts of *E*. *coli* ecotin or PBS (as a control) and the relative fluorescence units (RFU) were measured in 5 min intervals over a time frame of 60 min.

#### The FRET peptide

Terminally labelled fluorophore/quencher (FRET) peptide (Dabcyl-DQNATIDGRKQ-Edans, Edans-fluorophore and Dabyl-quencher)) carrying protease cleavage sits (i.e. IDGR) were custom ordered from GenScript, Inc. Peptides were resuspended in 10% isopropanol in deionized water to a final concentration of 573 μM and stored at -20°C until use. Peptides and reactions containing peptides were protected from light and wrapped in aluminum foil at all times unless stated otherwise.

#### The FRET peptide reaction

In an opaque 96 well plate Corning), 15 nM of ecotin protein from each species were mixed with 1 μl of FRET peptide and 20 μl of 10x factor Xa buffer in a total volume of 200 μl. The samples were analyzed in a plate reader with an excitation wavelength of 355 nm and emission of 530 nm as follows: the first scan was blanked and then 5 nM of factor Xa was added to the corresponding wells. Fluorescence was measured every 5 minutes over a timeframe of 1 h. A schematic of the FRET assay is depicted in Fig 4A.

### Neutrophil elastase inhibition assay

The efficiency of ecotin to inhibit NE was determined using the 96-well plate-based Fluorometric NE-Activity Assay Kit (BioVision #K383-100) according to the instructions of the manufacturer. The kit utilizes the ability of NE to proteolytically cleave a proprietary synthetic substrate to release an antibody-fluorophore conjugate that can be quantified by fluorescence microplate readers. First the Michaelis constant (K_m_) for NE was determined. At this concentration the readout (fluorescence) was in the linear range over a time frame of 15 min at 37°C. Protection assays were then carried out in triplicate with 25 nM NE, ecotin concentrations ranging from 0 nM to 50 nM and 25 nM of NE substrate in a final volume of 50 μl. Samples were measured in a microplate reader for fluorescence at excitation 380 nm and emission 500 nm. Data were fitted using the equation described in [51]: *v* = V_∞_ + (V_0_ –V_∞_)/(1 + 10^(log[Ecotin] − logIC50)^) using a non-linear curve fitting [52] as implemented by the program GraphPad Prism (GraphPad software), were *v* is the measured rate, V_∞_ is the rate at infinite inhibitor concentration, V_0_ is the rate at zero inhibitor concentration, and IC_50_ is the concentration of inhibitor required to produce half-maximal inhibition. In all cases the fitted value for V_∞_ was close to zero, indicating that there was little background hydrolysis of the peptide. Results were plotted as the logarithm to the base 10 of the IC_50_ for each ecotin protein.

### Isolation of human neutrophils and serum

Whole blood was drawn from healthy adult volunteers at the Health Center or the Clinical Translational Research Unit of the University of Georgia under informed consent according to procedures approved by the Institutional Review Boards at the University of Georgia (UGA# 2012-10769-06). For serum preparations, 10 ml of blood was drawn into a silicone coated tube and allowed to clot at room temperature for 30 min. The cellular components settled to the bottom while the pinkish supernatant containing some remaining cells was aspirated from the top, centrifuged at 10,000 xg, 5 min and cleared by filtration (0.22 μm). Autologous serum was kept on ice to prepare assay media for neutrophil killing assay and opsonization of bacteria. Polymorphonuclear leukocytes (PMNs) were purified as previously described [53]. Briefly, red blood cells were removed by dextran sedimentation of the anticoagulant-treated blood (35–40 ml) and neutrophils were separated using multistep Percoll gradient centrifugation. The purity of the preparations resulted in more than 97% neutrophils (cytospin) and cell viability was higher than 99% (Trypan Blue dye exclusion).

### Neutrophil killing assay

Bacterial killing by human neutrophils was determined as described [53]. Isolated neutrophils were washed two times with 1 mL assay medium (1 x HBSS containing 1% (v/v) autologous serum, 5 mM glucose, 10 mM HEPES) and adjusted to 9 x 10^6^ neutrophils/ml. Bacteria were washed two times with 1 x PBS and adjusted to 1 x 10^8^ cells/ml. 90 μl of bacteria were then mixed with 10 μl autologous serum of each neutrophil donor and incubated at room temperature for 5 minutes. Subsequently, purified human neutrophils and washed, serum-opsonized bacteria were mixed at a ratio of 10:1 multiplicity of infection (MOI, bacteria:neutrophil) in 1.5 ml Eppendorf tubes and incubated at 37°C with shaking (200 rpm). Aliquots from each sample (30 μl) were taken at 0, 10, 20 minutes of incubation, diluted 100-fold with 1xHBSS containing 1 mg/ml saponin and kept for 5 min on ice to lyse neutrophils and release live but ingested bacteria. Bacterial cell/saponin solutions were further diluted (100-fold with 1x HBSS) to decrease the saponin concentration. Samples were kept on ice until all aliquots were processed. 40 μl of each assay solution was then transferred to a fresh 96-well plate containing 160 μl of LB medium/well. Plates were pre-incubated at 37°C for 10 minutes before bacterial growth was followed in an EON microplate reader (BioTek) at OD_600_ over a time period of 8 h with measurements taken at two min intervals. Initial bacterial concentrations were determined using standards composed of samples with known bacterial concentrations. Bacterial killing was defined as decrease in the number of surviving bacteria compared to time zero.

### Preparation of neutrophil extracellular traps

The NET release assay and collection was performed according to a previously published protocol [54]. Briefly, neutrophil extracellular traps (NETs) were prepared by stimulating purified human neutrophils seeded in a 96-well microplate with 100 nM phorbol-myristate-acetate (PMA) for 4–6 h. NET formation was confirmed visually by observing characteristic morphological changes in neutrophils via light microscopy. Following stimulation, the supernatant of neutrophils containing secreted soluble molecules was very carefully removed and replaced by an equivalent volume of sterile 1 x HBSS (NETs that remained attached to the bottom of the wells). Next, NETs were subjected to limited DNAse digestion (1 U/ml DNAse I (Sigma Aldrich), 15 minutes) as previously described [54]. DNAse activity was stopped by adding 1 mM EGTA. The contents from the microwells were then collected in Eppendorf tubes and were centrifuged at 1,000 x g to remove cells and cell debris. Supernatants containing NETs were defined as NETs and stored at -80°C until use. NETs were purified from neutrophils obtained from 11 independent healthy controls and were subsequently pooled and used in the described experiments. The DNA content of each “NET prep” was determined by Quant-iT™ PicoGreen™ dsDNA Assay Kit (ThermoFisher) following the manufacturer’s instructions. The DNA concentration of NET preparations ranged from 0.91 to 4.81 ng/μl. The myeloperoxidase concentration of the NET preparations was quantitated with the Human Myeloperoxidase DuoSet ELISA kit (R&D Systems) and ranged from 39.2 to 388.7 ng/ml. NE concentrations in NETs were determined by the Recombinant Human NE/ELA2 Protein ELISA kit (R&D Systems) and ranged from 32.5 to 266.7 ng/ml. Although they only provide relative quantitation, non-commercial ELISA kits were also performed as previously described [54,55] and also detected NET-specific MPO-DNA and NE-DNA complexes in the NET preparations used in this study.

### Bacterial killing by NETs

The bactericidal activity of NETs was determined by colony counting. First, 50 μl of bacterial suspension was prepared as described above and adjusted to a concentration 1 x 10^7^ cells/ml and mixed with 150 μl of NETs (prepared as above) or with 150 μl of 1x PBS in a 96 well plate. After incubation for 30 min at 37°C, a 10-fold dilution series (in 1x PBS) was prepared, 10 μl volume from each step was plated onto LB agar plates and incubated at 37 ^o^C until single colonies were visible and countable. Results were expressed as CFU/ml.

### Statistical analyses

Results were analyzed by one-way ANOVA with an ad-hoc Dunnett's or pairwise *t*-test. Each experiment was independently performed at least three times and PMNs were isolated from different donors. Differences with a *p* value < 0.05 were considered significant.

## Results

### Identification and *in silico* analyses of *Campylobacter* ecotin orthologs

**Fig 1:**
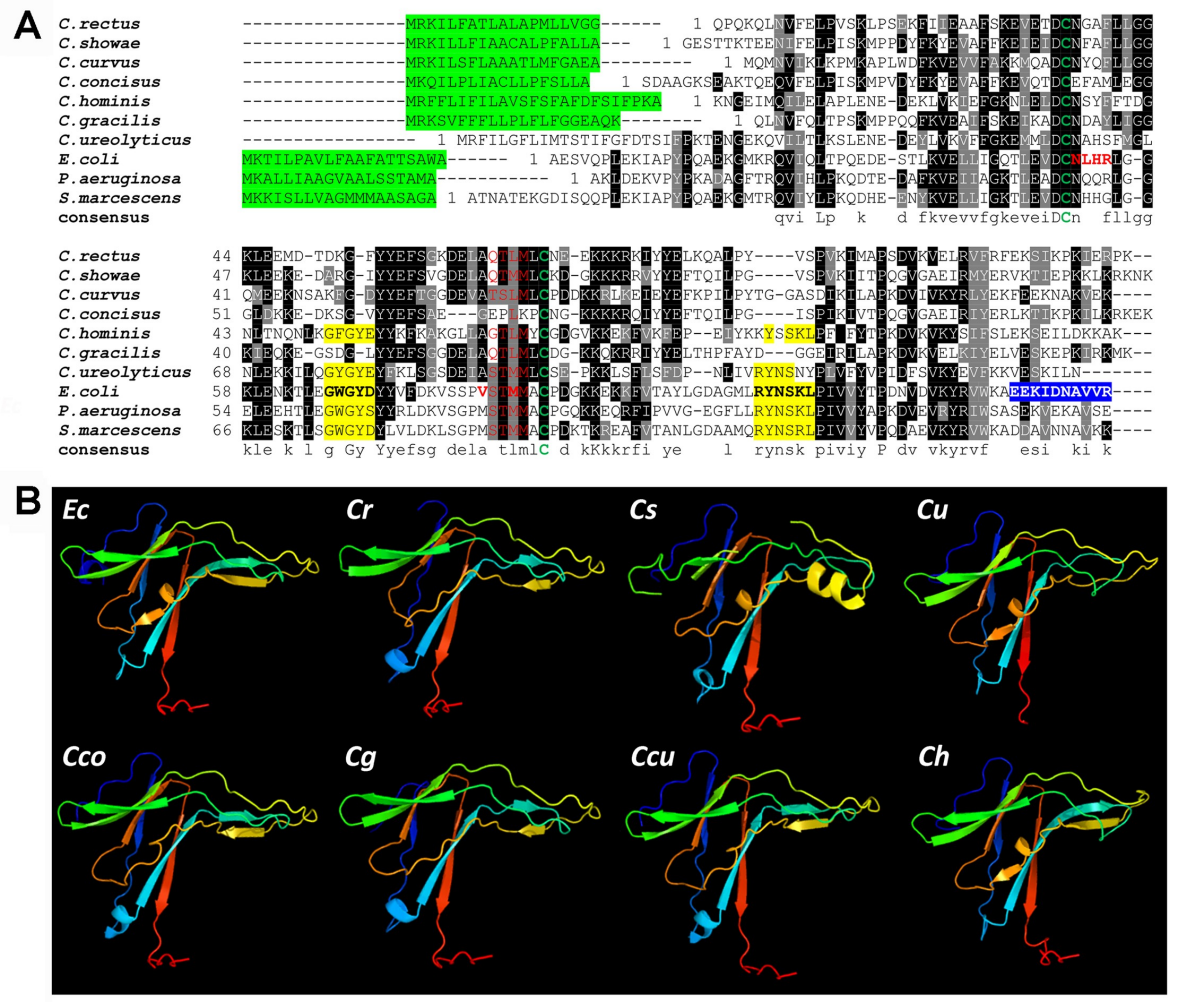
Sequence comparison and secondary structural analysis of ecotins from *Campylobacter* spp. **(A)** A multiple sequence alignment was generated with ClustalW and BOXshade; http://www.ch.embnet.org/ with ecotin sequences from *C*. *rectus*, WP_004320172.1; *C*. *showae*, WP_002949877.1; *C*. *cur*vus, WP_011991897.1; *C*. *concisus*, WP_107709881.1, *C*. *hominis*, WP_012108817.1, *C*. *gracilis*, WP_005871804.1*; C*. *ureolyticus*, WP_016646581.1; *E*. *coli* (WP_137532711.1) and other species (*P*. *aeruginosa*, WP_132540651.1, *S*. *marcescens*, WP_015671699.1. Black shading indicates >50% amino acid identity. Grey shading is >50% similarity in amino acid charge. In red, the primary protease binding sites (including the P1 residue (*Ec-*Met84) and residues 51–54; in yellow, the secondary protease binding sites (*Ec*-residues 67 to 70 and 108 to 113); green letters, conserved cysteine residues; in blue, dimerization interface (*Ec*-residues 133 to 142) [59–61]; highlighted in green, signal peptide according to SignalP [47] (cut-off 0.5, except for *C*. *ureolyticus*, here no signal peptide was predicted even with a cut-off of 0.3). **(B)** *In silico* structural analysis of ecotins using the Protein Homology/analogY Recognition Engine V2.0 (PHYRE2) is shown. Ecotin proteins from *E*. *coli* (*Ec*), *C*. *rectus* (*Cr*), *C*. *showae*, (*Cs*), *C*. *ureolyticus* (*Cu*), *C*. *concisus* (*Cco*), *C*. *gracilis* (*Cg*), *C*. *curvus* (*Ccu*) and *C*. *hominis* (*Ch*) display very similar structures despite the low % of amino acid conservation between the *E*. *coli* and the *Campylobacter* homologs (i.e. ecotins from *C*. *rectus*, *C*. *showae*, *C*. *ureolyticus*, *C*. *curvus*, *C*. *gracilis*, *C*. *concisus*, *C*. *hominis* share 27%, 33%, 37%, 34%, 31%, 25% and 39% amino acid identity with the *E*. *coli* ecotin, respectively).

*In silico* analysis of the *pgl* gene cluster of *Campylobacter rectus* previously identified a 429 nt open reading frame (CAMRE0001_2237) in a three gene locus located between the protein-N-glycosylation genes *gne* and *pglK* [56], encoding a protein with 27% homology to the ecotin protein from *E*. *coli* (Fig 1A). To gain insight into the conservation of this ecotin homolog among *Campylobacter* species, homology searches against the protein sequence database (BlastP, [57]) using either the amino acid sequence of the *C*. *rectus* or the *E*. *coli* ecotin as a query, revealed that potential ecotin proteins were also present in *C*. *showae*, *C*. *cur*vus, *C*. *concisus*, *C*. *hominis*, *C*. *gracilis* and *C*. *ureolyticus*. Interestingly, the ecotin gene was found upstream of the *pgl* operon only in *C*. *rectus*, *C*. *showae* and *C*. *curvus*. Despite the low conservation between proteins at the amino acid level (e.g. *C*. *rectus* and *C*. *showae* ecotins only show 27% and 33% identity to the *E*. *coli* homolog, respectively), further structure prediction using PHYRE2 [58] resulted in 100% confidence of the ecotin-fold in these *Campylobacter* species when compared to the *E*. *coli* protein (Fig 1B).

Further analyses of the ecotin structures revealed that the two cysteines, Cys50 and Cys87 (that form an intra-subunit disulfide bond in *E*. *coli* ecotin) [59,60], are conserved in the *Campylobacter* homologs. In the substrate binding pocket, the P3 (*Ec*-Ser82) and P4 (*Ec*-Val81) residues are different when compared *E*. *coli*, however, those sites are somewhat conserved among the *Campylobacter* proteins while the P2 site (*Ec*-Thr83) is conserved in 5 out of 7 *Campylobacter* ecotins. Interestingly, the methionine at the P1 site (*Ec*-Met84), responsible for directly targeting the active site of the serine protease [62], is only present in the *C*. *showae* protein, while the ecotins from *C*. *rectus* and other *Campylobacters* harbor a leucine in this position (Fig 1A). Based on these differences and similarities between the *E*. *coli*, the *C*. *show*ae and *C*. *rectus* ecotins, we characterized the homologous proteins from these two oral *Campylobacter* species in more detail.

### Expression and purification of ecotin

**Fig 2:**
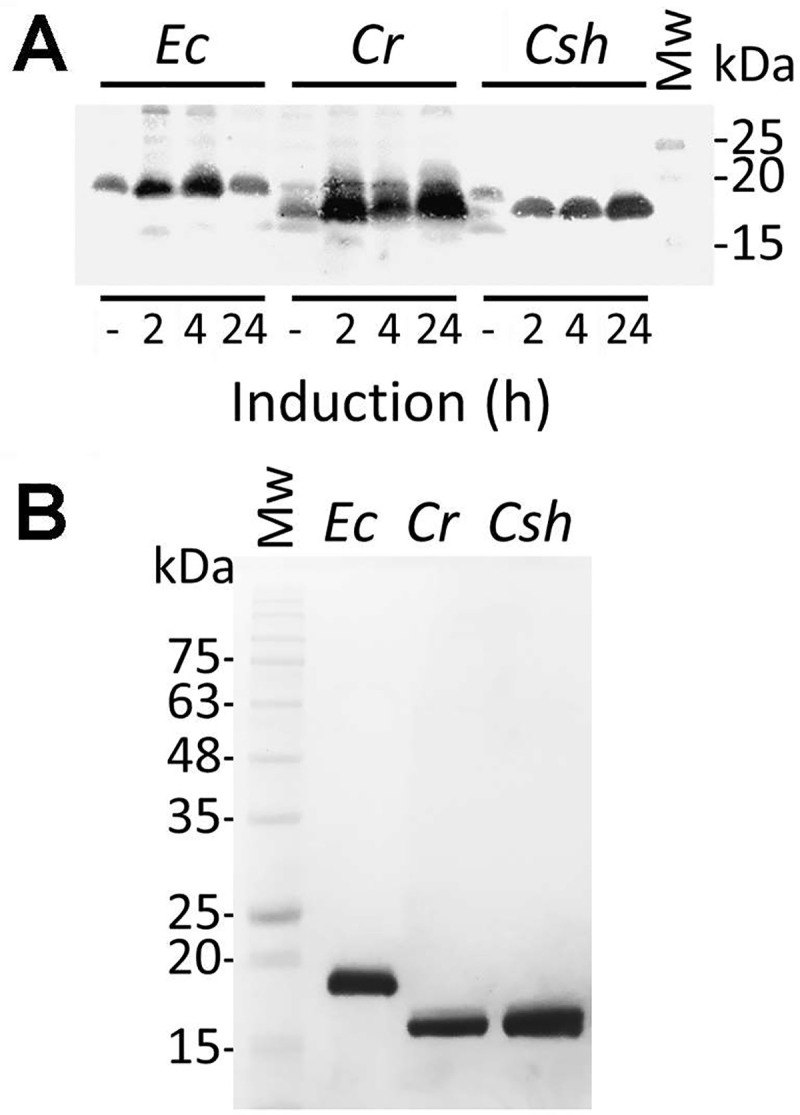
Overexpression and purification of *Campylobacter* ecotin in *E*. *coli*. **(A)** Western blot with hexa-histidine-specific antibodies of whole cell lysates to follow the expression of *Campylobacter* ecotins in *E*. *coli* BL21 after 2, 4 and 24 h of induction with IPTG is shown, protein samples before induction (-) were included as controls. The signal migrating at ~18 kDa represents the ecotin-His_6_ protein from the indicated species, *Ec*, *E*. *coli*; *Cr*, *C*. *rectus*; *Csh*, *C*. *showae*. A full, top to bottom scan of the membrane is provided in the supplement, S1 Fig. **(B)** SDS-PAGE (15%, Coomassie stained) of ecotin proteins from the indicated species after overexpression and purification from whole cell lysates of *E*. *coli* BL21. Molecular weight markers (Mw, in kDa) are indicated on the left.

To investigate their protease inhibition properties, the ecotins from *C*. *rectus*, *C*. *showae* and *E*. *coli* were expressed as C-terminal 6xHisTag fusion proteins and purified from *E*. *coli*. The *E*. *coli* ecotin protein is a periplasmic protein [30]; however, secretion signal predictions using SignalP revealed a low probability for such a peptide in the *Campylobacter* ecotins. Therefore, we replaced the predicted native signal peptide (Fig 1A) with the *pelB* secretion signal located on plasmid pET22b. Analysis of whole cell lysates by western blotting after induction with IPTG at different time points is shown in Fig 2A and a full scan of the western blot is shown in the supplement (S1 Fig). Ecotin proteins were purified from *E*. *coli* whole cell lysates by Ni-NTA chromatography after 24 h of induction (Fig 2B).

### Ecotin inhibits trypsin-mediated protein degradation

**Fig 3:**
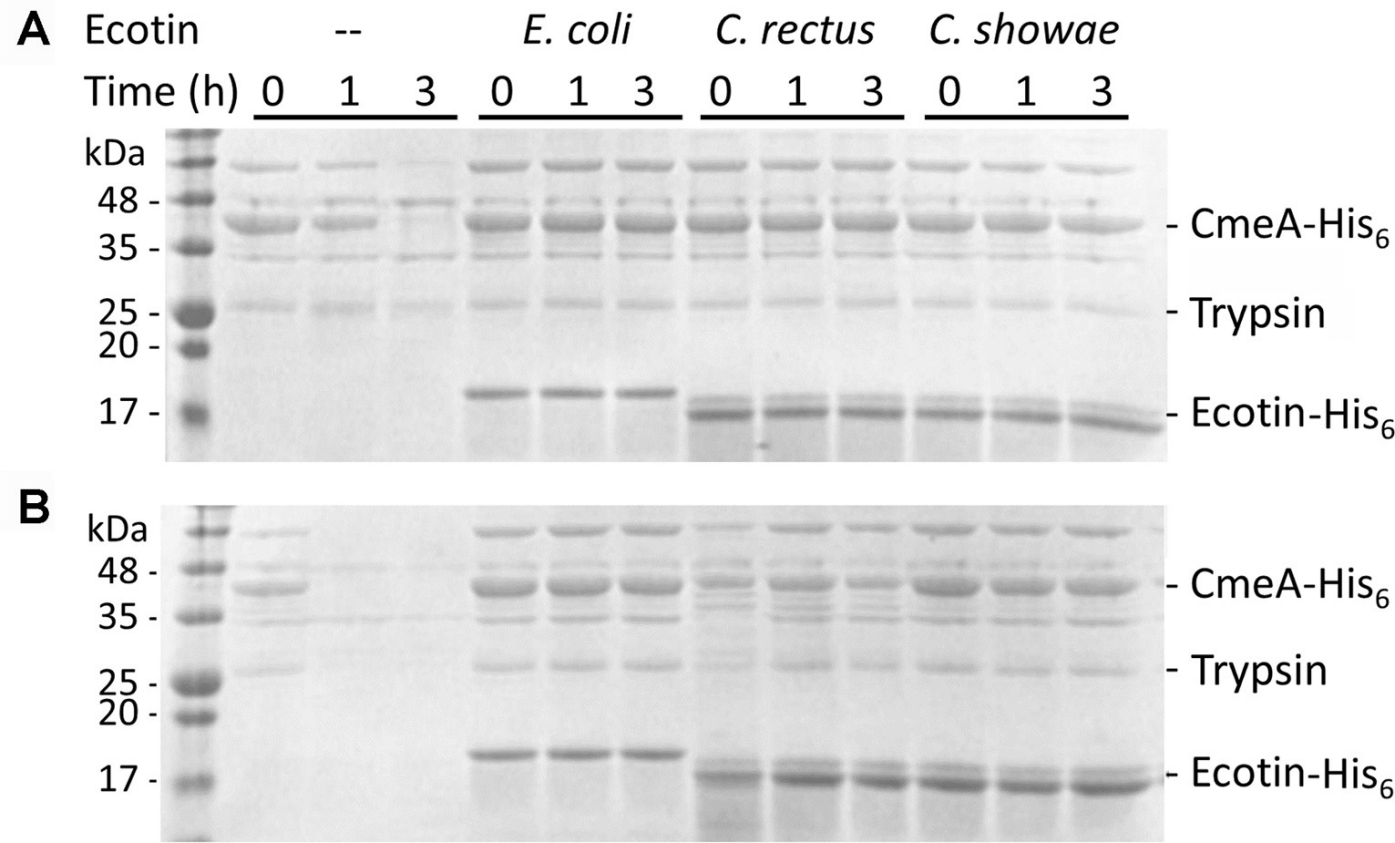
Ecotins inhibit trypsin-mediated proteolysis. SDS-PAGE (15%, Coomassie stained) of trypsin protease protection assays by ecotin proteins carried out at: **(A)** 37°C and **(B)** 45°C are shown. Samples contained the protease substrate CmeA-His_6_ (10 nM), trypsin (10 nM) and ecotin (15 nM) with the indicated strain. (—) indicates the absence of ecotin from the assay. Aliquots were taken at t = 0, and after 1 h and 3 h of incubation. The signals migrating at ~18 kDa represent the ecotin-His_6_ proteins; the signals migrating at ~42 kDa represent CmeA-His_6_. Molecular weight markers (in kDa) are indicated on the left. Full scans of the gels are available in the supplement (S2 Fig).

Purified ecotin proteins from *C*. *showae* and *C*. *rectus* were tested for their ability to inhibit the serine protease, trypsin. Ecotin from *E*. *coli*, previously shown to inhibit this protease [30], was used as a control (Fig 3 and S2 Fig). In the absence of ecotin, complete degradation of the *C*. *jejuni* multidrug efflux pump protein (CmeA) was observed within 1 h of incubation at 45°C and within 3 h of incubation at 37°C. This indicated that CmeA partially unfolds at the higher temperature, potentially exposing trypsin sites that are less accessible at 37°C. In the presence of ecotin from either *E*. *coli*, *C*. *rectus*, or *C*. *showae*, no CmeA degradation could be observed at 37°C or 45°C over the duration of the assay indicating that the *Campylobacter* ecotin homologs are indeed active in inhibiting trypsin (Fig 3 and S2 Fig). No CmeA degradation occurred in the absence of proteases (S3 Fig).

### Ecotin does not confer protection against self-proteases HtrA and DegP

Next, we investigated the potential of ecotins to inhibit the self-protease DegP from *E*. *coli* and the corresponding homolog from *C*. *jejuni* HtrA (S3 Fig). DegP- and HtrA-mediated proteolysis resulted in approximately 50% CmeA degradation after 3 h incubation, with no intact CmeA detectable after 6 h incubation indicating that both DegP and HtrA are active after expression and purification from *E*. *coli*. However, ecotin from *E*. *coli*, *C*. *rectus*, or *C*. *showae* had no effect on the proteolytic activities of HtrA and DegP (S3 Fig). No CmeA degradation could be observed after prolonged incubation at 37°C or 45°C in the absence of DegP/HtrA, verifying that the protein substrate is stable over the time frame of the assay.

### FRET assay to measure ecotin protection against factor Xa

**Fig 4:**
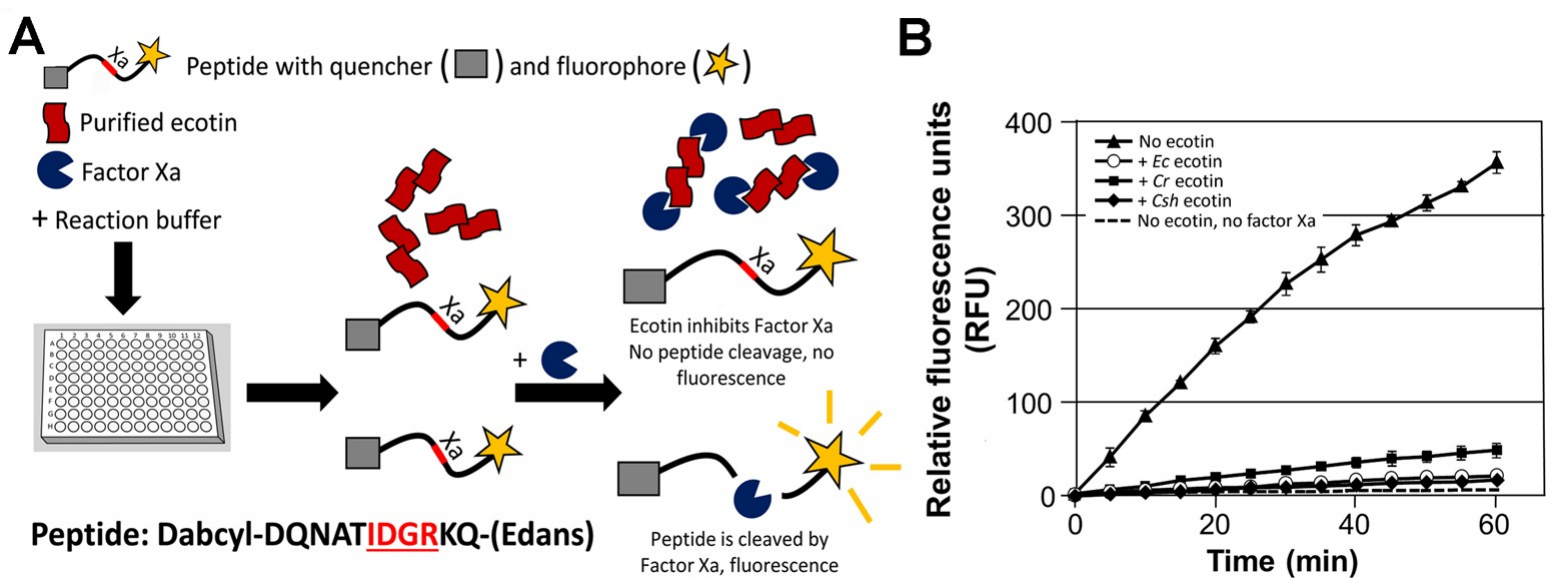
Ecotins inhibit factor Xa-mediated FRET peptide cleavage. **(A)** Illustration of the *in vitro* FRET assay. FRET peptides were incubated with purified ecotin homologs in 96-well plates with or without factor Xa. When the peptide is cleaved by the protease, fluorescence is produced. The factor Xa cut-site (IDGR) in the FRET peptide is highlighted in red. **(B)** The FRET peptide was incubated with factor Xa (5 pmol) and ecotin (15 nM) from *E*. *coli* (open circles), *C*. *rectus* (filled squares) and *C*. *showae* (filled diamonds) over a time frame of 60 min in 96 well plates. The control wells contained FRET peptide substrate only (no ecotin, filled triangles), the basal fluorescence level (no ecotin, no factor Xa) is indicated by a dashed line. Arbitrary fluorescent units (y-axis) were determined using a microplate reader with a filter set of Ex/Em = 355/530 nm. Standard deviations are indicated by error bars.

In this study, we have adapted (based on the work of [63]) a high-throughput fluorescence resonance energy transfer (FRET) 96-well plate-based assay to investigate the inhibitory properties of ecotin from *E*. *coli*, *C*. *rectus* and *C*. *showae* against factor Xa (Fig 4A). In the absence of ecotin, 350 relative fluorescent units (RFU) were observed after 60 min of incubation with factor Xa indicating cleavage of the peptide (Fig 4B), Control reactions without factor Xa maintained a basal fluorescence level of less than 10 RFU from the beginning to the end of the incubation period indicating that the peptide had not been degraded over the duration of the assay. Addition of *E*. *coli* ecotin resulted in a significant reduction in RFUs (21.3 ± 1.5) after 60 min of incubation when compared to the absence of ecotin, indicating that little to no cleavage of the peptide by factor Xa had occurred; this clearly demonstrated that the *E*. *coli* ecotin effectively inhibits factor Xa under these experimental conditions. Addition of *C*. *rectus* or *C*. *showae* ecotins also resulted in a significant reduction of RFUs indicating that the ecotin homologs from the oral *Campylobacter* species also target factor Xa. While the inhibition by the *C*. *showae* ecotin was similar to the *E*. *coli* homolog and resulted in 17.7 ± 3.2 RFU after 60 min of incubation, addition of the *C*. *rectus* ecotin resulted in significantly higher (48 ± 8.2) RFUs (Fig 4B) when compared to the *E*. *coli* and *C*. *showae* proteins indicating that this ecotin variant has a lower potency for inactivating factor Xa.

### Ecotin inhibits the serine protease neutrophil elastase

Ecotin proteins were further tested for their ability to inhibit NE. First, we determined that the K_m_ for NE was 27.61 ± 11.84 nM (S4A Fig) and that the linearity of the assay ranges from t = 0 to t = 15 minutes (S4B Fig). Here, an increase in background fluorescence subtracted RFU from 0 (t = 0 min) to 6519 ± 38 (t = 15 min) clearly indicated the cleavage of the substrate by NE.

**Fig 5:**
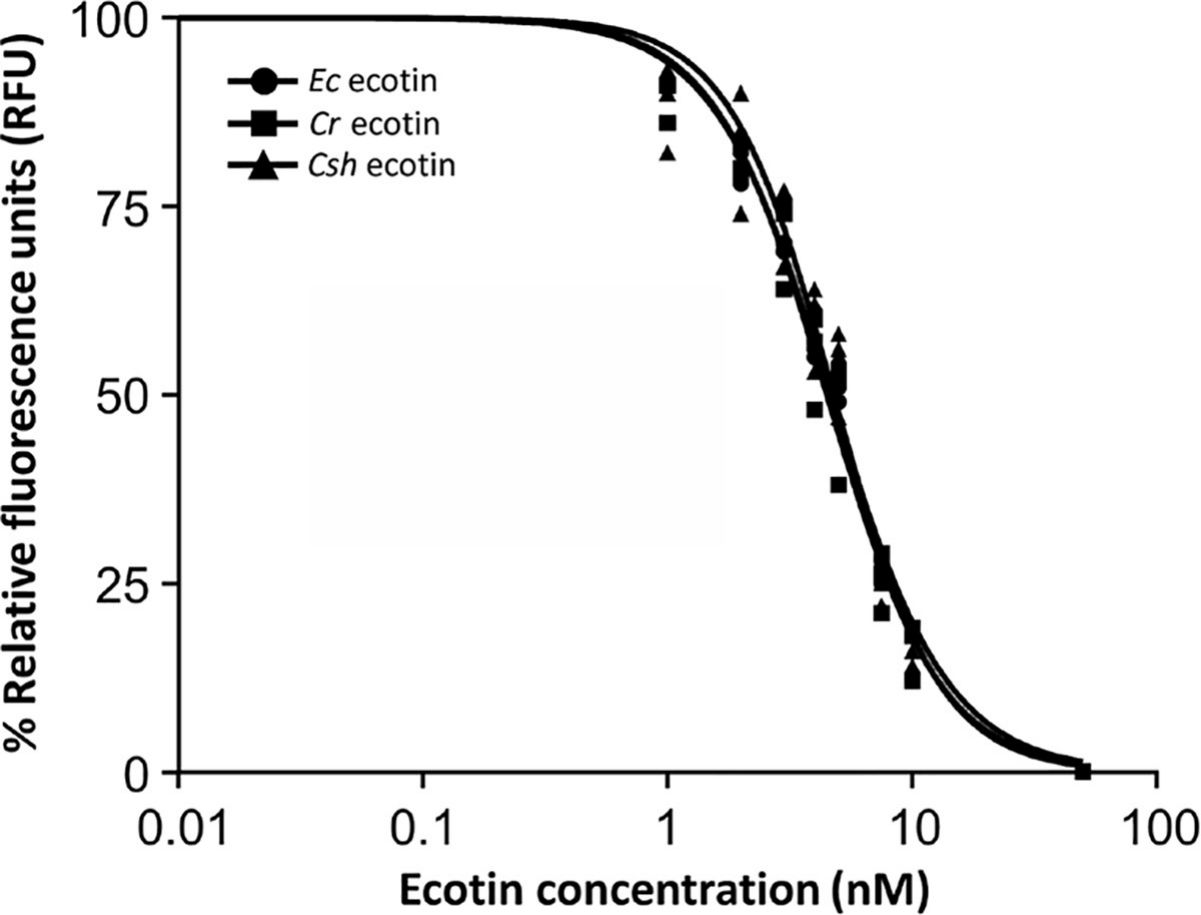
*E*. *coli* and *Campylobacter* ecotins possess similar IC_50_ values for neutrophil elastase. Experimentally determined IC_50_ values (nM) for *E*. *coli* (circles, 4.64 ± 0.23), *C*. *rectus* (squares, 4.49 ± 0.25) and *C*. *showae* (triangles, 4.78 ± 0.31) ecotins used at increasing concentrations (nM) to inhibit NE. Each data point represents the mean from three independent measurements. Relative florescence (in %, where 100% indicates fully digested FRET-peptide and 0% indicates fully inhibited protease) was determined after measuring the samples in a microplate reader with a filter set of Ex/Em = 355/530 nm. Separate graphs for each IC_50_ determination were included in the supplementary information (S5 Fig).

Next, the inhibitory properties of the three ecotin proteins were evaluated. In the absence of ecotin, 1.2x10^4^ RFU were observed after 15 min of incubation while addition of *E*. *coli* ecotin resulted in a concentration-dependent inactivation of NE indicated by lower RFUs with increasing protein amounts (0 nM to 50 nM). Fitting of the titration curves resulted in an IC_50_ for the *E*. *coli* ecotin protein towards elastase of 4.64 ± 0.23 nM (Fig 5 and S5A Fig). Similar results were observed when the inhibitory properties of *C*. *rectus* and *C*. *showae* ecotin homologs were monitored. Here, the IC_50_ for elastase was 4.49 ± 0.25 nM for the *C*. *rectus* and 4.78 ± 0.31 nM for the *C*. *showae* ecotin (Fig 5 and S5B & S5C Fig). Therefore, it can be concluded that both *Campylobacter* ecotin proteins have very similar inhibitory properties for NE (no significant difference, one-way ANOVA, *p* > 0.05) when compared to the *E*. *coli* ecotin.

### *Campylobacter* ecotin proteins protect *E*. *coli* cells from killing by live human neutrophils and purified NETs

**Fig 6:**
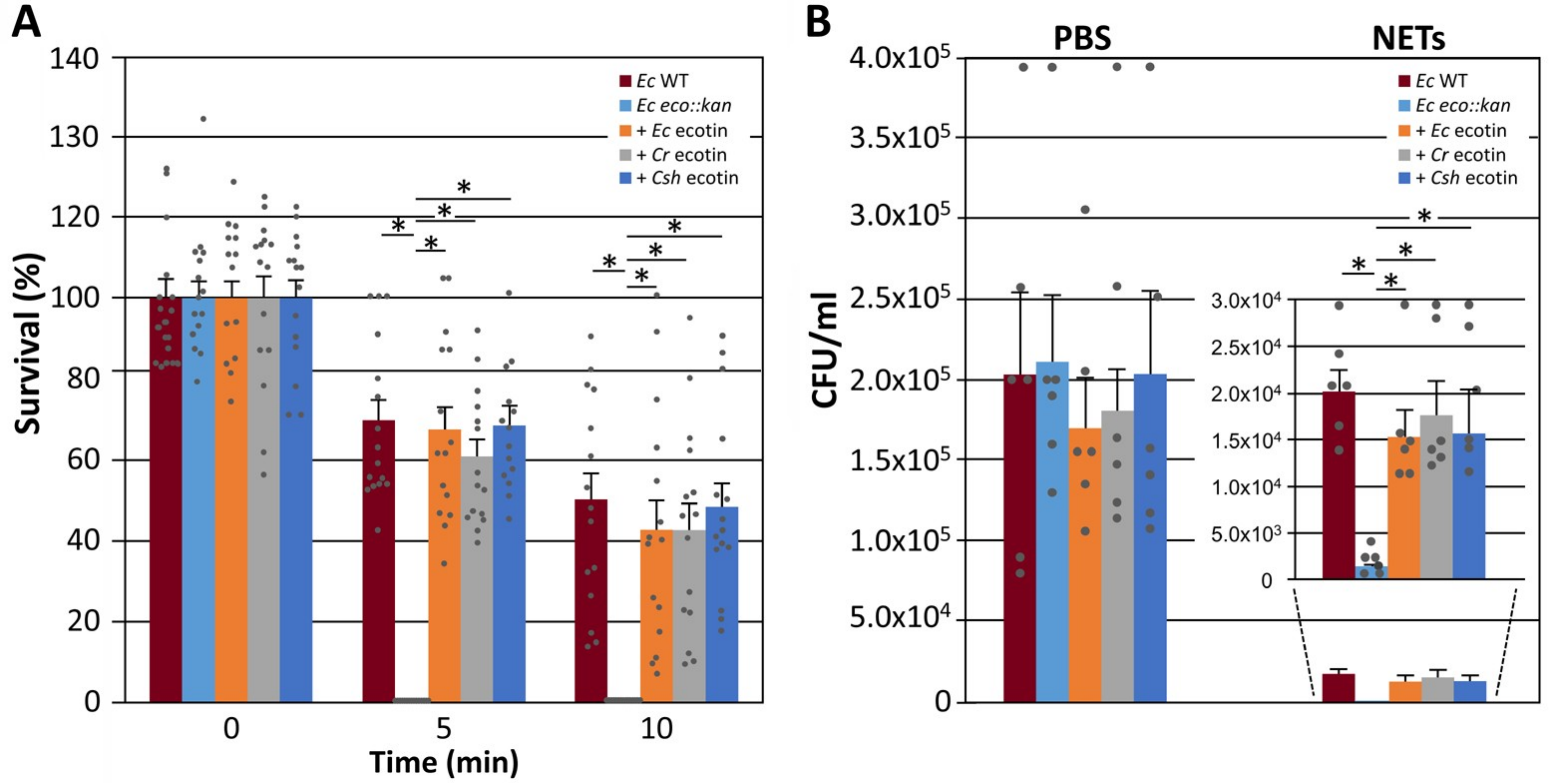
Campylobacter ecotins rescue neutrophil-mediated killing of ecotin-deficient E. coli and protect E. coli cells from killing by live and purified NETs. ***(*A)** The results of a time-dependent neutrophil killing assay are shown. *E*. *coli* BL21 WT (*Ec-*WT), the corresponding *E*. *coli* BL21 ecotin mutant (*Ec eco*::*kan*), and *Ec eco*::*kan* complemented with either the *E*. *coli* (*+ Ec* ecotin), *C*. *rectus* (*+ Cr* ecotin) or *C*. *showae* ecotin (*+ Csh* ecotin) were incubated with human neutrophils and bacterial survival was determined using a microplate-based bacterial growth assay. Remaining bacteria (expressed in % survival, based on colony forming units CFU in each sample (100% = 1 x 10^7^ bacteria) were calculated based on a CFU per OD_600_ standard curve that was created for each strain (not shown). Error bars represent the standard deviation for a dataset obtained from 3 biological replicates (each done in triplicate) using neutrophils from different human donors. **(B)** Bar graph of colony counts determined from LB agar plates after spotting 10 μl of 10-fold serial dilution series of cells of the indicated *E*. *coli* strains after 30 min of incubation with PSB (control) or NETs (the original plate pictures from 3 biological replicates are shown in S7 Fig). The insert depicts the values for the NET samples at a different scale; strain designations are identical to (A). Error bars depict the standard error of the mean (SEM). Statistically significant differences (paired t-test, *p*<0.005) are indicated by an asterisk.

To assess if *Campylobacter* ecotins when expressed *in trans* in an *E*. *coli* ecotin-deficient mutant can prevent killing by live human neutrophils, a high throughput 96-well microplate-based bacterial survival assay was employed. After 5 min of incubation with human neutrophils, 100% (1 x10^7^ CFU) of the *E*. *coli* ecotin mutant bacteria were killed, whereas the *E*. *coli* wild-type showed a statistically significant increase in survival (70% or 7 x10^6^ CFU remaining) (Fig 6A). Complementation with the native *E*. *coli* ecotin and the *C*. *show*ae ecotin resulted in similar average survival rates of 67% and 68% respectively. Expression of ecotin from *C*. *rectus* resulted in a slightly lower, but also significantly increased average survival rate of 60%. After 10 min of incubation with human neutrophils, an average survival rate of 50% was observed for the *E*. *coli* wild-type while human neutrophils incubated with the *E*. *coli* ecotin mutant complemented with ecotins from *E*. *coli*, *C*. *rectus* and *C*. *showae* showed slightly (but not statistically significant) lower average survival rates of 42%, 42% and 48% respectively. All *E*. *coli* strains showed a 100% survival rate in the absence of human neutrophils (S6 Fig).

The ability of ecotin to protect intact cells of *E*. *coli* was further evaluated in killing assays with purified NETs isolated from human neutrophils. NE is associated with the DNA scaffold in NETs stimulated by several stimuli such as Gram-negative bacteria, including *C*. *jejuni* [3,64,65]. NE remains enzymatically active in NETs and could expose entrapped bacteria to proteolytic damage [64,66,67]. Our results indicate that NETs are capable of reducing *E*. *coli* WT numbers by 1-log compared to the PBS control, corresponding to 90% growth reduction. However, when *E*. *coli* ecotin mutant was mixed with NETs, a significantly lower number of cells were remaining (0.1% survival equivalent to 1.0x10^4^ cells) after 5 min of incubation, that is an additional 1-log decrease relative to the *E*. *coli* WT and a 2-log decrease compared to the PBS control (Fig 6B and S7 Fig). This indicates that ecotin provides some, but not full protection against NETs. Expression of either the native *E*. *coli* ecotin or the ecotin homologs from *C*. *rectus* or *C*. *showae* resulted in a statistically significant increase in average survival rates when compared to the ecotin mutant (Fig 6B and S7 Fig). Those survival rates were similar when compared to the *E*. *coli* WT indicating that the *Campylobacter* and *E*. *coli* ecotin proteins possess similar protective properties against killing by purified NETs.

### Ecotins from *C*. *rectus* and *C*. *showae* partially protect *C*. *jejuni* cells from proteases present in chicken cecal contents

Ecotin expression was investigated in whole cell lysates of *C*. *jejuni* 81–176 WT and the *pglB* mutant carrying the C-terminal His_6_-tagged *E*. *coli* ecotin, or the native or *pelB*-fused ecotins from *C*. *rectus* and *C*. *showae* on plasmid pCE107-28 (S8 Fig). Western blot analysis revealed that in comparison to the control (*Ec*-ecotin expressed in *E*. *coli*), the *E*.*coli* variant was not expressed in the *C*. *jejuni* WT or the *pglB* mutant. Similar results were obtained for the *pelB*-fusion of the two *Campylobacter* ecotins (S8 Fig). In contrast the native version of the *C*. *rectus* and *C*. *showae* ecotins could be detected with His_6_-specific antibodies in whole cell lysates of *C*. *jejuni* WT and the *pglB* mutant; however, the corresponding signals were absent in whole cell lysates of the *E*. *coli* control strain indicating that the native versions are not expressed in this background (S8 Fig).

**Fig 7:**
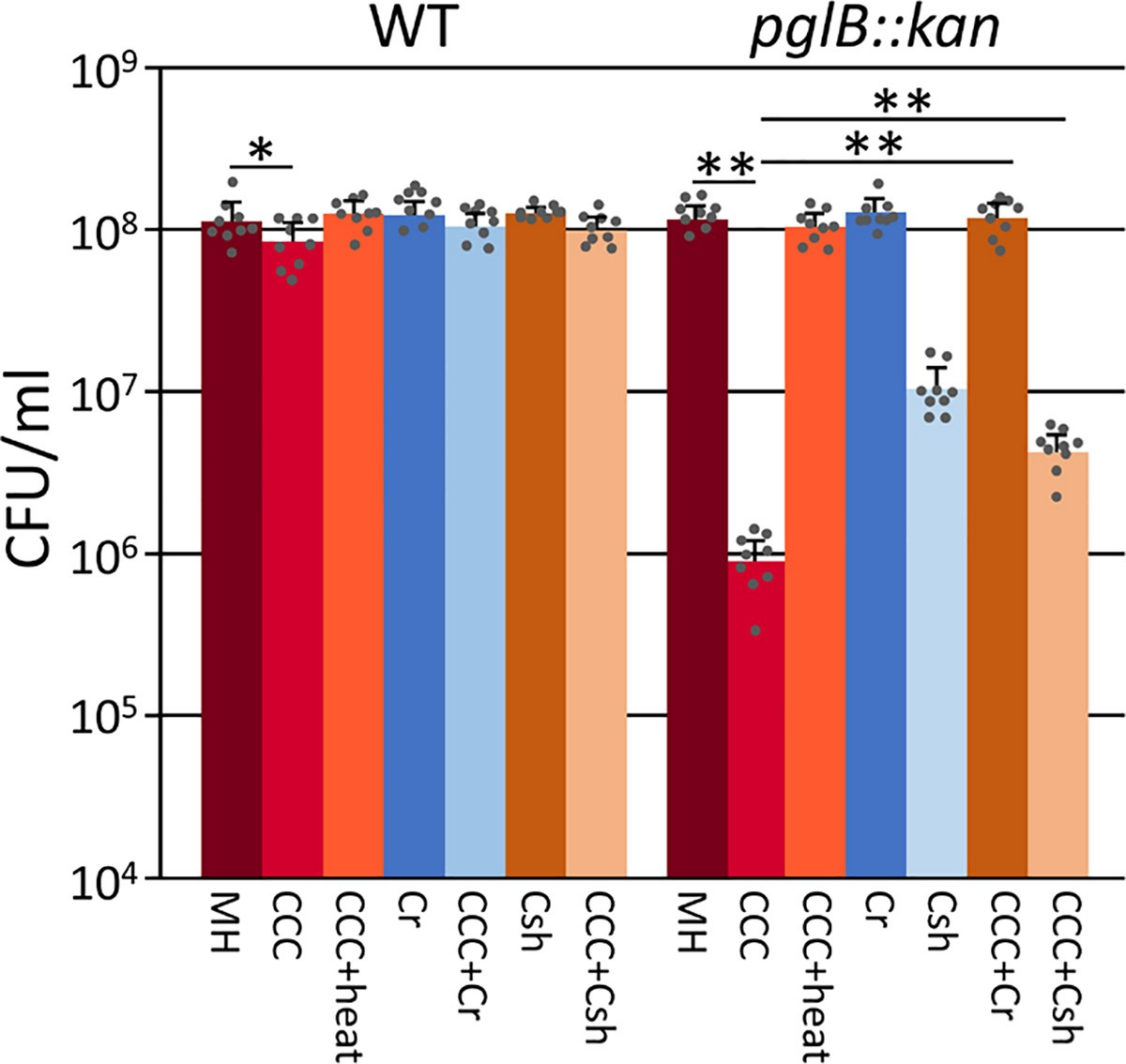
Expression of *C*. *rectus* and *C*. *showae* ecotins partially rescue the protease sensitive phenotype of a *C*. *jejuni* N-glycosylation mutant. CFU of the *C*. *jejuni* wildtype and the *C*. *jejuni pglB* mutant expressing native ecotins from *C*. *rectus* (*Cr*) or *C*. *showae* (*Csh*) were determined in media supplemented with chicken cecal contents (CCC). CFU determined in the absence of CCCs and heat inactivated CCCs were used as controls. Bars represent the mean from 3 biological replicates carried out as triplicates; standard deviations are indicated by error bars, statistical differences (one-way ANOVA) between the control (MH) and the experimental samples (Dunnett's test) as well as in between samples (paired *t*-test) are indicated (*p<0.05, **p<0.005).

Since N-linked protein glycosylation in *C*. *jejuni* has been implicated in protection from proteases present in chicken cecal contents (CCC) [13], we aimed to investigate if the ecotins from the oral *Campylobacters* can rescue the loss of N-glycosylation phenotype and reverse the associated higher susceptibility to proteases present in the chicken gut (Fig 7). As previously described [13], we found that survival of the *pglB* mutant was significantly decreased when compared to the control (*p***<**0.005). Interestingly, the WT also showed a slight but statistically significant (*p* **=** 0.04) reduced survival rate when incubated with CCC from 1 week old chicks. Expression of both the *C*. *rectus* and the *C*. *showae* ecotins resulted in a statistically significant increase in *C*. *jejuni pglB* survival when compared to survival of the *pglB* mutant not expressing ecotin (*p*<0.005) indicating the partial neutralization of proteases present in CCC (Fig 7). Expression of both ecotins in the *C*. *jejuni* WT also indicated a partial reduction of the protease effect; however, the increase was not statistically significant. Expression of *C*. *rectus* and *C*. *showae* ecotins had no effect on the survival of the WT and the *pglB* mutant (in the absence of CCC). Similarly incubation with heat inactivated CCC resulted in comparable survival rates that were obtained in the absence of CCC.

## Discussion

In this study, we have identified and characterized ecotin homologs from two oral *Campylobacter* species. In *C*. *rectus* and *C*. *showae*, the corresponding open reading frames are located upstream of the protein glycosylation locus. N-glycosylation of proteins is ubiquitous in *Campylobacter* spp. [17] and has been implicated in numerous cellular functions [56], including protection against the intestinal proteases for the enteric pathogen, *C*. *jejuni* [13]. In contrast, *C*. *rectus* is a recognized oral pathogen implicated in causing periodontitis [20], while *C*. *showae* has been linked to causing gingivitis and periodontitis, and more recently with inflammatory bowel disease [68]. For these oral pathogens, ecotin may provide an additional layer of protection against the proteolytic attack of tissue-specific serine proteases, and in rare cases against gastric and pancreatic enzymes (e.g. trypsin) that could reach the oral cavity [69], particularly if those oral *Campylobacters* also trigger NET release and potentially induce the movement and infiltration of neutrophils to infection sites as has been shown for *C*. *jejuni* [65,70,71].

Indeed, the ecotin homologs from *C*. *rectus* and *C*. *showae* behave similar to the *E*. *coli* ecotin in their ability to inhibit trypsin, factor Xa, and NE, with comparable IC_50_ values for the latter protease. It is worth mentioning that our IC_50_ values were two-fold higher in comparison to previously described values determined for the *E*. *coli* inhibitor [51], however, differences in assay conditions including temperature and protein purification/storage protocols may contribute to this discrepancy. The potency of ecotin to inhibit NE is emphasized by its IC_50_ that is within the range of some synthetic, pre-clinically and clinically tested NE inhibitors [72], that have IC_50s_ in the low nM or even pM range [73,74]. Moreover, ecotins are more potent than most natural compounds that have IC_50s_ in the mid to low μM range [75,76], however some of them, like soybean Kunitz trypsin inhibitors (SKTIs) inhibit NE with IC_50_ values as low as 0.3 nM [77]. None of the tested ecotins, in this study, were active in preventing protein degradation by bacterial self-proteases HtrA from *C*. *jejuni* or DegP from *E*. *coli*. This supports previous observations that ecotins only protect against exogenous proteases [31] and is likely due to the ability of the HtrA/DegP proteins to form barrel shaped proteasomes preventing ecotins from entering and inhibiting the active sites within these HtrA/DegP oligomers [78].

To further investigate ecotin function, we determined whether the ecotins could compensate for loss of N-glycosylation in the related gastrointestinal pathogen, *C*. *jejuni*, and its increased susceptibility towards proteases present in chicken cecal contents [13]. Partial rescue upon expression of the native *C*. *rectus* and *C*. *showae* proteins indicated that the ecotins were able to partially neutralize the proteases present in CCC. This is consistent with ecotin inhibition being limited to serine proteases of the trypsin/chymotrypsin fold while metalloproteases, also detected in the chicken gut [13], are not substrates for this inhibitor [79]. In addition, an ecotin-deficient *E*. *coli* strain was complemented with ecotin homologs from *C*. *rectus* and *C*. *showae*. When incubated with intact neutrophils, the serine protease NE is proposed to attack and cleave the *E*. *coli* outer membrane protein A (OmpA), allowing NE access into the periplasm where it digests periplasmic and inner membrane proteins resulting in loss of cell viability and inhibition of growth [31,80]. Both *Campylobacter* ecotins were able to rescue the *E*. *coli* ecotin-deficient mutant at a level that was comparable to the native *E*. *coli* ecotin. This suggests that *E*. *coli* and *Campylobacter* ecotins possess a similar inhibitory mechanism where ecotins form head to tail homodimers and tightly bind up to two protease molecules through the formation of a hetero-tetramer [31,59,62,81]. However, based on the amino acid variations between the *E*. *coli* and *Campylobacter* ecotins (Fig 1A), the similarities in their protease-inhibitory properties were surprising. In general, the ability to inhibit a wide range of proteases is derived from two active sites. The primary active site contains hydrophobic amino acids that can bind the catalytic triad of many serine-proteases. The primary binding site includes 4 additional residues (Ec-51-54) that interact with trypsin [59]. The secondary binding site is derived from the second ecotin molecule that binds non-specifically to the target, thus providing additional affinity for the protease [36,59,61]. These two points of contact mechanisms result in a strong binding affinity for a broad range of proteases. Interestingly, the secondary binding site is not conserved between the *E*. *coli* and the two *Campylobacter* proteins and one prominent residue, the “one-size fits all” methionine at the P1 site, described to be important for the broad specificity of the inhibitor in *E*. *coli* [62,82], is altered to a leucine in *C*. *rectus* (and other *Campylobacters*). However, it has also been shown that certain amino acid exchanges in P1-Met84 have no effect on the inhibition of trypsin (some even result in an increase of the K_i_) whereas the affinity for elastase was dramatically reduced in some (in e.g. Met84Lys), but not in other variants (e.g. Met84Ile) [83,84]. Moreover, while wild-type ecotin does not inhibit thrombin, factor XIa, activated protein C, and plasmin, Met84Arg or Met84Leu mutants can bind and inhibit these proteases with relatively high affinity [29,85]. Therefore, variations in affinities of *Campylobacter* ecotins towards other, untested proteases cannot be ruled out at this point and warrants further investigation.

In summary, the presented data suggest that ecotin may play a key role in the survival of *C*. *rectus* and *C*. *showae* in the oral cavity of mammalian hosts. It is currently unknown why certain *Campylobacter* species acquired (or retained) ecotin homologs while the thermophilic species, including *C*. *jejuni*, that typically inhabit the gastrointestinal tract do not. It is tempting to speculate that oral *Campylobacter* species may have evolved this second line of defense in addition to their N-glycosylation systems to protect themselves against proteases, like NE, the most prominent serine protease in this niche.

## Supporting information

S1 Fig Overexpression of *Campylobacter* ecotin in *E*. *coli*. (DOCX)

S2 Fig Ecotins inhibit trypsin-mediated proteolysis. (DOCX)

S3 Fig Ecotin does not protect from proteolytic degradation by the self-proteases DegP and HtrA. (DOCX)

S4 Fig V_max_ and K_m_ for neutrophil elastase. (DOCX)

S5 Fig *E*. *coli* and *Campylobacter* ecotins possess similar IC_50_ values for neutrophil elastase. (DOCX)

S6 Fig *Campylobacter* ecotins rescue neutrophil-mediated killing of ecotin-deficient *E*. *coli*. (DOCX)

S7 Fig *Campylobacter* ecotins protect *E*. *coli* cells from killing by live and purified NETs. (DOCX)

S8 Fig Expression of ecotins in *Campylobacter*. (DOCX)

S1 Table Oligonucleotides used in this study. (DOCX)
