# Supplementary material for: Characterization of ecotin homologs from Campylobacter rectus and Campylobacter showae
Source: PLoS One. 2020 Dec 30;15(12):e0244031. doi: 10.1371/journal.pone.0244031 (PMC7773321; doi:10.1371/journal.pone.0244031)
Supplement: S1 Fig — (DOCX) [file pone.0244031.s001.docx]

**Figure S1**

**Figure S1. Overexpression of *Campylobacter* ecotins in *E. coli*.** Full top-to-bottom scan of the membrane shown in Fig. 2A of the main manuscript. Western blot with hexa-histidine-specific antibodies of whole cell lysates to follow the expression of *Campylobacter* ecotins in *E. coli* BL21 after 2, 4 and 24 h of induction with IPTG is shown. Protein samples before induction (-) were included as controls. The signal migrating at ~18 kDa represents the ecotin-His_6_ protein from the indicated *Campylobacter* species. Relevant molecular weight markers (Mw, in kDa) are indicated on the right. Western blot image was captured using a Canon MF4700 scanner in combination with the Canon MF Toolbox 4.9.
