# Supplementary material for: Characterization of ecotin homologs from Campylobacter rectus and Campylobacter showae
Source: PLoS One. 2020 Dec 30;15(12):e0244031. doi: 10.1371/journal.pone.0244031 (PMC7773321; doi:10.1371/journal.pone.0244031)
Supplement: S2 Fig — (DOCX) [file pone.0244031.s002.docx]

**Figure S2**

**
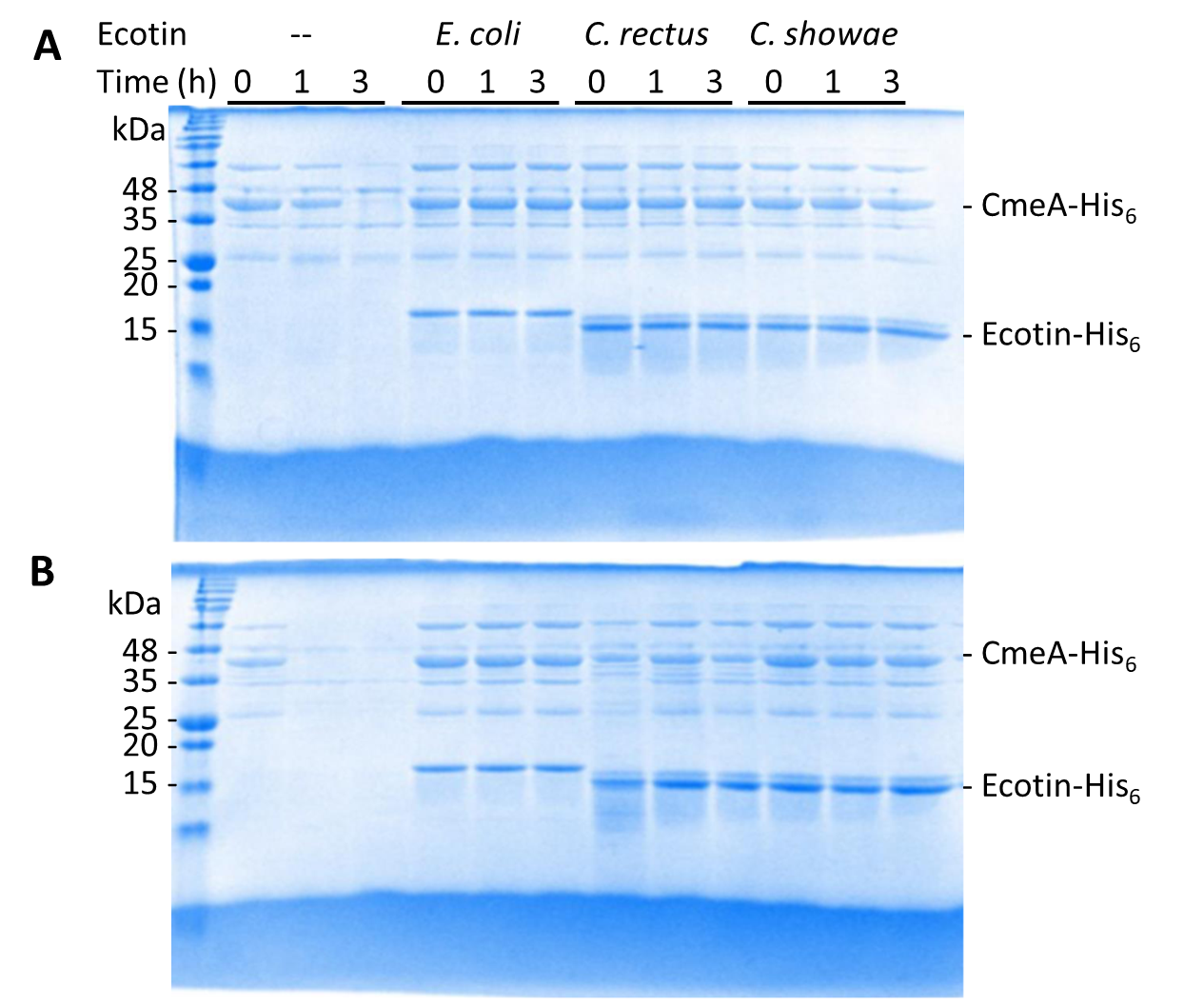
**

-Trypsin

-Trypsin

**Figure S2. Ecotins inhibit trypsin-mediated proteolysis.** Full top-to-bottom scans of 15% SDS-PAGE Coomassie stained gels shown in Fig 3. Trypsin protease protection assays with ecotin proteins carried out at **(A)** 37^o^C and **(B)** 45^o^C are shown. Samples contained the protease substrate CmeA-His_6_ (10 nM), trypsin (10 nM) and ecotin (15 nM) with the indicated strain. (--) indicates the absence of ecotin from the assay. Aliquots were taken at t=0, and after 1 h and 3 h of incubation. The signals migrating at ~18 kDa represent the ecotin-His_6_ proteins. The signals migrating at ~42 kDa represent CmeA-His_6_. Molecular weight markers (in kDa) are indicated on the left. Protein gel image was captured using the Bio-Rad Gel Doc XR+ Gel Documentation System in combination with the Image Lab 6.1 Software.
