# Supplementary material for: Characterization of ecotin homologs from Campylobacter rectus and Campylobacter showae
Source: PLoS One. 2020 Dec 30;15(12):e0244031. doi: 10.1371/journal.pone.0244031 (PMC7773321; doi:10.1371/journal.pone.0244031)
Supplement: S3 Fig — (DOCX) [file pone.0244031.s003.docx]

**Figure S3**

**
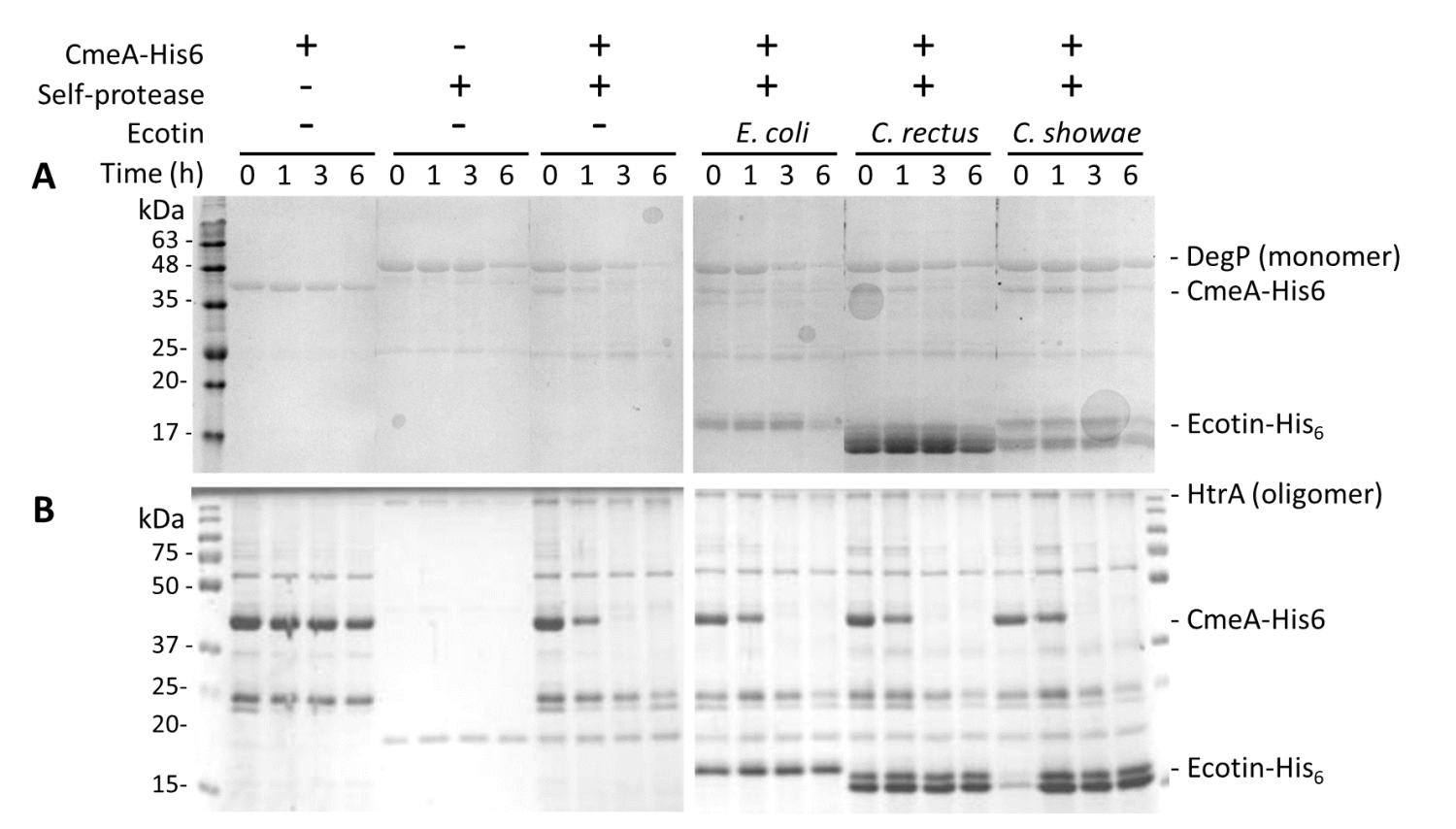
**

**Figure S3. Ecotin does not protect from proteolytic degradation by the self-proteases DegP and HtrA.** Coomassie stained 12.5% SDS-PAGE gels of **(A)** *E. coli* DegP and **(B)** *C. jejuni* HtrA self-protease protection assays by ecotin proteins are shown. Samples contained 1 nM CmeA, 1 nM DegP or HtrA and 15 nM ecotin (as indicated). The absence or presence of each component is indicated by (-) or (+), respectively. Aliquots were taken at t=0, and after 1, 3 and 6 h of incubation. he signals migrating at ~18 kDa represent the ecotin-His_6_ proteins, the signals migrating at 42 kDa represent CmeA-His_6_, the monomeric form of DegP-His_6_ migrates at 54 kDa and HtrA-His_6_ (52 kDa) migrates as an oligomer (most likely as a hexamer) at a higher molecular weight. Molecular weight markers (in kDa) are indicated on the left. Protein gel images were captured using the Bio-Rad Gel Doc XR+ Gel Documentation System in combination with the Image Lab 6.1 Software.
