# Supplementary material for: Characterization of ecotin homologs from Campylobacter rectus and Campylobacter showae
Source: PLoS One. 2020 Dec 30;15(12):e0244031. doi: 10.1371/journal.pone.0244031 (PMC7773321; doi:10.1371/journal.pone.0244031)
Supplement: S4 Fig — (DOCX) [file pone.0244031.s004.docx]

**Figure S4**

**
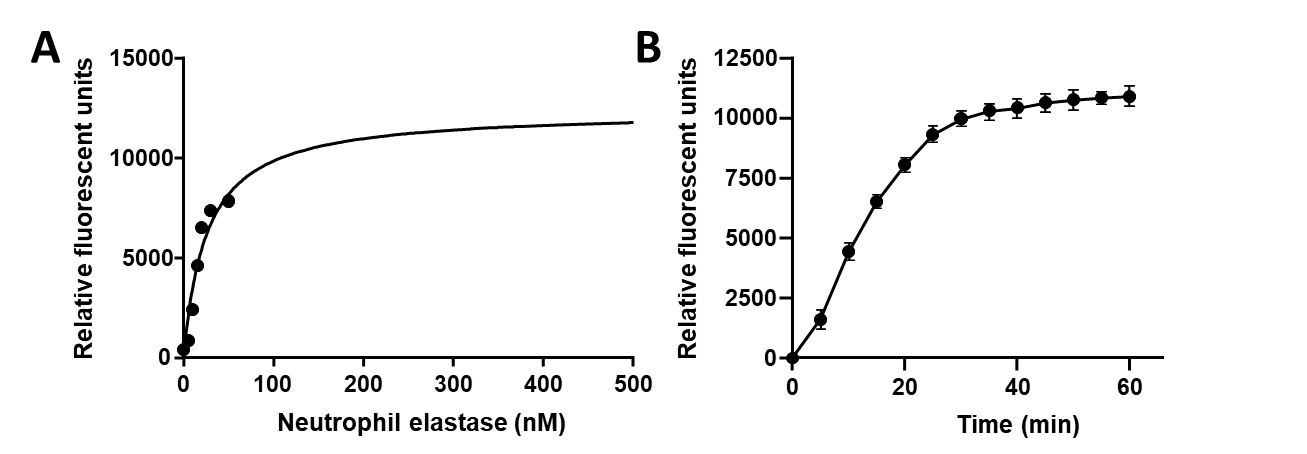
**

**Figure S4. V_max_ and K_m_ for neutrophil elastase. (A)** The Michaelis-Menten graph of neutrophil elastase is shown. The K_m_ (27.61 ± 11.84) and V_max_ (12430 ± 2098) were calculated using the Michaelis-Menten equation following graphing the results of enzymatic activity obtained with different substrate concentrations (in nM). **(B)** Time-dependent neutrophil elastase assays were performed to determine the linear range of the enzyme at the K_m_ of 25 nM. The readout indicated by background subtracted arbitrary fluorescent units was measured using a microplate reader with a filter set of Ex/Em = 355/530 nm. Each data point represents the average value from three independent measurements. Standard deviations are indicated by the error bars.
