# Supplementary material for: Characterization of ecotin homologs from Campylobacter rectus and Campylobacter showae
Source: PLoS One. 2020 Dec 30;15(12):e0244031. doi: 10.1371/journal.pone.0244031 (PMC7773321; doi:10.1371/journal.pone.0244031)
Supplement: S5 Fig — (DOCX) [file pone.0244031.s005.docx]

**Figure S5**

**
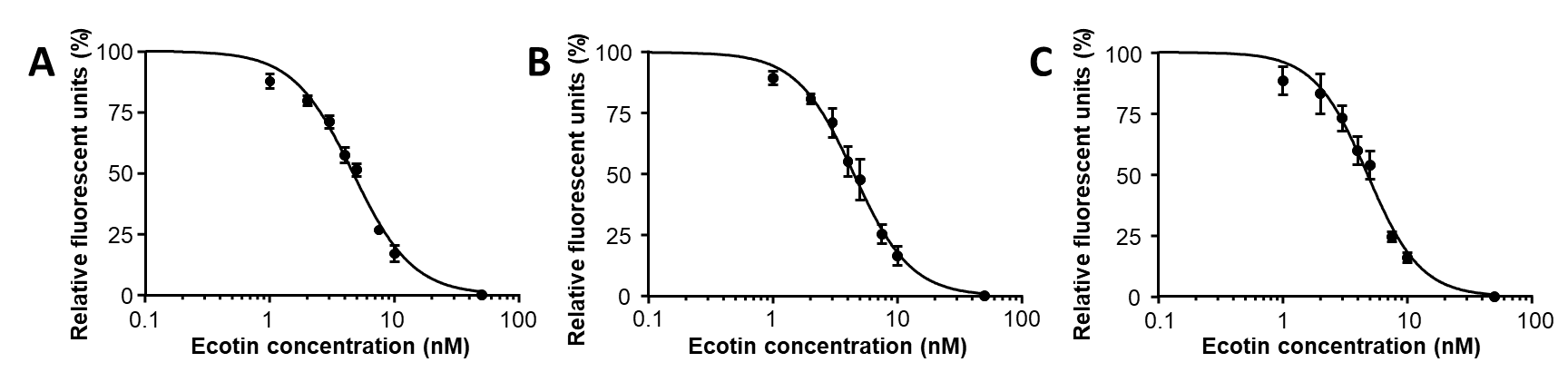
**

**Figure S5. *E. coli* and *Campylobacter* ecotins possess similar IC_50_ values for neutrophil elastase.** Graphs (for Figure 5) for the experimentally determined IC_50_ values (nM) are shown for **(A)** *E. coli* (4.64 ± 0.23)*,* **(B)** *C. rectus* (4.49 ± 0.25), and (**C)** *C. showae* (4.78 ± 0.31) ecotins used at the indicated concentrations (in nM) to inhibit neutrophil elastase. Each data point represents the mean from three independent experiments. Standard deviations are indicated by error bars. Relative fluorescence of the samples (in %, where 100% indicates fully digested FRET-peptide and 0% indicates fully inhibited protease) was determined in a microplate reader with a filter set of Ex/Em = 355/530 nm.
