# Supplementary material for: Characterization of ecotin homologs from Campylobacter rectus and Campylobacter showae
Source: PLoS One. 2020 Dec 30;15(12):e0244031. doi: 10.1371/journal.pone.0244031 (PMC7773321; doi:10.1371/journal.pone.0244031)
Supplement: S6 Fig — (DOCX) [file pone.0244031.s006.docx]

**Figure S6**

**
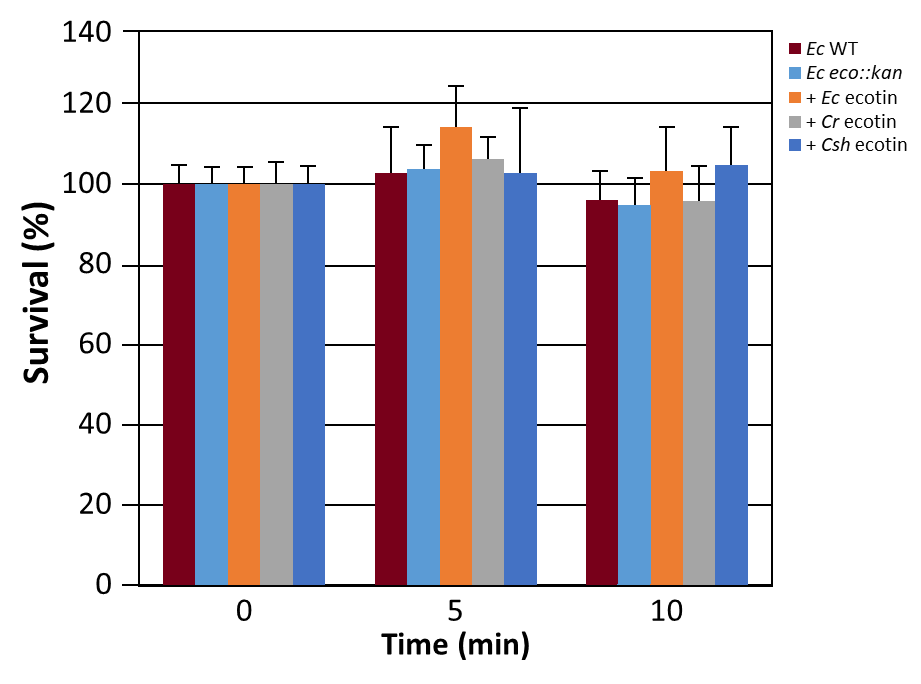
**

**Figure S6. *Campylobacter* ecotins rescue neutrophil-mediated killing of ecotin-deficient *E. coli***. The results for the control (absence of human neutrophils) for the time-dependent neutrophil killing assay (manuscript, Fig. 6A) are shown. *E. coli* BL21 WT (*Ec* WT), the corresponding ecotin mutant (*Ec eco::kan*), and the *E. coli* BL21 ecotin mutant complemented with either the *E. coli* (+ *Ec* ecotin), *C. rectus* (+ *Cr* ecotin) or *C. showae* (+ *Csh* ecotin) ecotins were incubated in the absence of human neutrophils and bacterial survival was determined using a microplate-based bacterial growth assay. Remaining bacteria (expressed in % survival, based on colony forming units (CFU) in each sample (100% = 1 x 10^7^ bacteria) were calculated based on a CFU per OD_600_ standard curve that was created for each strain. Error bars depict the standard error of the mean (SEM). No significant difference (one-way ANOVA, *p* > 0.05) was observed between strains over the time frame of the assay.
