# Supplementary material for: Characterization of ecotin homologs from Campylobacter rectus and Campylobacter showae
Source: PLoS One. 2020 Dec 30;15(12):e0244031. doi: 10.1371/journal.pone.0244031 (PMC7773321; doi:10.1371/journal.pone.0244031)
Supplement: S7 Fig — (DOCX) [file pone.0244031.s007.docx]

**
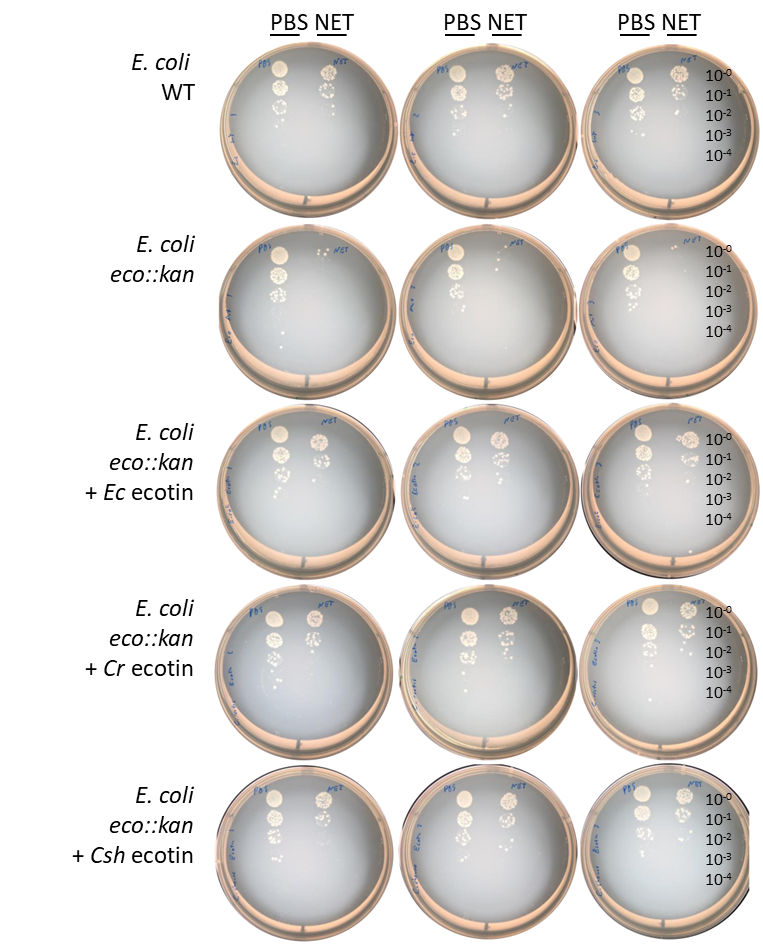
Figure S7**

**Figure S7. *Campylobacter* ecotins protect *E. coli* cells from killing by live and purified NETs*.*** LB agar plates (in triplicate) after spotting 10 µl of 10-fold serial dilution series of cells of the indicated *E. coli* strain after 30 min incubation with PBS (control) or neutrophil NET supernatant solutions are shown. Colony counts were used to generate the bar graph (Fig. 6B) in the manuscript.
