# Supplementary material for: Characterization of ecotin homologs from Campylobacter rectus and Campylobacter showae
Source: PLoS One. 2020 Dec 30;15(12):e0244031. doi: 10.1371/journal.pone.0244031 (PMC7773321; doi:10.1371/journal.pone.0244031)
Supplement: S8 Fig — (DOCX) [file pone.0244031.s008.docx]

**Figure S8**

**
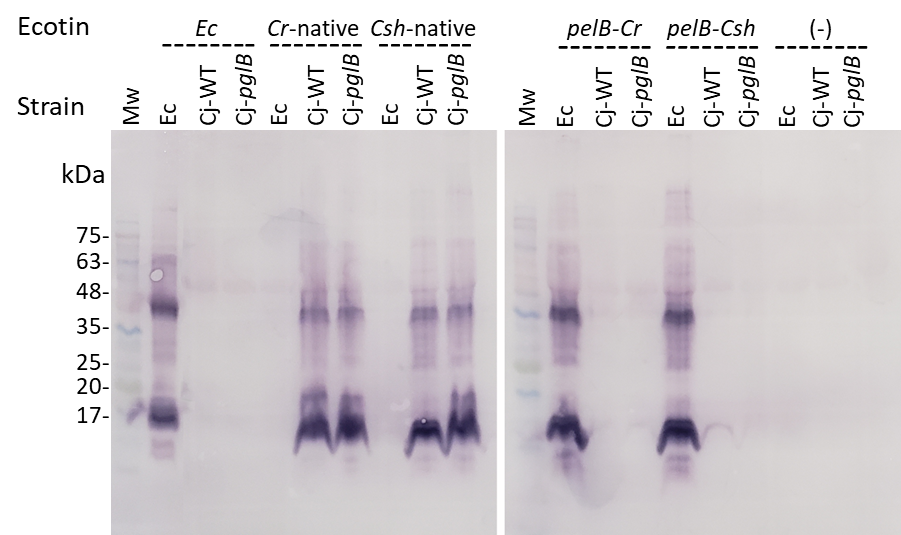
**

**Figure S8. Expression of ecotins in *Campylobacter.*** Western blot (full top-to-bottom scan) with hexa-histidine-specific antibodies of whole cell lysates of *E. coli* (*Ec*), *C. jejuni* wildtype (Cj-WT) and *C. jejuni* *pglB* mutant (Cj-*pglB*) after separation on a 12.5% SDS-PAGE gel and transfer to a PVDF membrane. The signal migrating at ~18 kDa represents either the ecotin-His_6_ protein from *E. coli* (*Ec*), or the native or *pelB* leader peptide fused ecotins from *C. rectus* (*Cr*) or *C. showae* (*Csh*). Whole cell lysates not expressing ecotin (-) were included as background signal controls. Relevant molecular weight markers (Mw, in kDa) are indicated on the left. Western blot image was captured using a Canon MF4700 scanner in combination with the Canon MF Toolbox 4.9.
