# Supplementary material for: Characterization of ecotin homologs from Campylobacter rectus and Campylobacter showae
Source: PLoS One. 2020 Dec 30;15(12):e0244031. doi: 10.1371/journal.pone.0244031 (PMC7773321; doi:10.1371/journal.pone.0244031)
Supplement: S1 Table — (DOCX) [file pone.0244031.s009.docx]

**Supplementary Information**

**Table S1.** Oligonucleotides used in this study.

| **Oligonucleotide** | **Sequence (5’ – 3’)** | ***Application*** |
| --- | --- | --- |
| pKD4-ecotin-F | ATGAAGACCATTCTACCTGCAGTATTGTTTGCCGCTTTCGTGTAGGCTGGAGCTGCTTCG | Create *E. coli* ecotin mutant |
| pKD4-ecotin-R | TTAGCGAACTACCGCGTTGTCAATTTTCTCTTCCGCCTTCATGGGAATTAGCCATGGTCC | Create *E. coli* ecotin mutant |
| EC-ecotin-F | TAACCTTCAGCGACATCATCGG | Confirm *E. coli* ecotin mutant |
| EC-ecotin-R | AACCGGCTCGGGCGTTGGATGTC | Confirm *E. coli* ecotin mutant |
| Ecotin-Cr-NdeI-F | TTAGTGAGCATATGAGAAAAATTTTATTTGCTACGTTGGCTTTAGCGCCGATGC, *Nde*I | Clone native ecotin of *C. rectus* |
| Ecotin-Cr-NcoI-F | ATATATCCATGGGGCCGCAAAAGCAACTAAACGTC, *Nco*I | Clone *pelB*- ecotin of *C. rectus* |
| Ecotin-Cr-XhoI-R | ATATCTCGAGTTTTGGCCTTTCTATTTTTGGTTTTATTGATTTTTCAAACC, *Xho*I | Clone ecotin of *C. rectus* |
| Ecotin-Csh-NdeI-F | TTAGTGAACATATGAGGAAAATTTTACTTTTTATCGCGGCTTGCGCGTTGCCG, *Nde*I | Clone native ecotin of *C. showae* |
| Ecotin-Csh-NcoI-F | ATATCCATGGGCGAAAGTACGACAAAAACCGAGGAAAATATTTTCGAGC, *Nco*I | Clone *pelB*- ecotin of *C.showae* |
| Ecotin-Csh-SalI-R | ATATGTCGACTTTATTTTTCCTTTTTAATTTTTTTGGTTCTATCG, *Sal*I | Clone ecotin of *C. showae* |
| Ecotin-Ec-NdeI-F | AGCAAACATATGAAGACCATTCTACCTGCAGTATTGTTTGCC, *Nde*I | Clone ecotin of *E. coli* |
| Ecotin-Ec-XhoI-R | ATATCTCGAGGCGAACTACCGCGTTGTCAATTTTCTCTTCCGCCTTCCAG, *Xho*I | Clone ecotin of *E. coli* |
| Ecotin-pET22b-BamHI-F | AATATGGATCCGGCGTAGAGGATCGAGATCTCG, *Bam*HI | Clone ecotins into pCE111-28 |
| Ecotin-pET22b-SalII-R | TAGCAGTCGACTCAGCTTCCTTTCGGGCTTTGTTAGC, *Sal*I | Clone ecotins into pCE111-28 |
| htrA-NdeI-F | ATTAAATCATATGAAAAAGATTTTTTTATCATTAAGTTTAGC, *Nde*I | Clone *htrA* of *C. jejuni* |
| htrA-XhoI-R | TTTCTCGAGTTTAAGCACAAGCAAAGTCGCAAAACC, *Xho*I | Clone *htrA* of *C. jejuni* |

Restriction sites are underlined.
